# Carboxylesterase 1 is a core metabolic vulnerability of platinum-resistant high-grade serous ovarian carcinoma

**DOI:** 10.1186/s13046-026-03766-8

**Published:** 2026-07-24

**Authors:** Irene Flati, Davide Vecchiotti, Fiorenza Di Giovanni, Daniela Verzella, Francesca Dall’Aglio, Angelo Sarra, Chiara Battistini, Melissa Zambuto, Giuseppe Frisullo, Jessica Cornice, Ilaria Nunzi, Mauro Di Vito Nolfi, Harry J. Whitwell, Francesca Veglianti, Hafiz Nadeem Khan, Saverio Marchi, Guido Franzoso, Ugo Cavallaro, Daniel D’Andrea, Edoardo Alesse, Francesca Zazzeroni, Daria Capece

**Affiliations:** 1 Department of Biotechnological and Applied Clinical Sciences (DISCAB), University of L’Aquila, L’Aquila, Italy; 2 Microscopy Center, University of L’Aquila, L’ Aquila, Italy; 3 Unit of Gynecological Oncology Research, European Institute of Oncology IRCCS, Milan, Italy; 4 Tumor Immunology and Immunotherapy Unit, IRCCS-Regina Elena National Cancer Institute, Rome, Italy; 5 Department of Immunology and Inflammation, Imperial College London, London, UK; 6 Department of Clinical and Molecular Sciences, Marche Polytechnic University, Ancona, Italy; 7 Department of Metabolism, Digestion and Reproduction, Division of Systems Medicine, Imperial College London, London, UK; 8 School of Engineering Mathematics and Technology, University of Bristol, Bristol, UK

**Keywords:** High Grade Serous Ovarian Carcinoma, Carboxylesterase 1, Metabolic adaptation, Autophagy, Chemoresistance

## Abstract

**Background:**

Platinum-resistant high-grade serous ovarian carcinoma (HGSOC) is a major clinical challenge, with high mortality rates and no effective therapeutic options. Metabolic adaptations—including enhanced lipid catabolism, oxidative phosphorylation (OXPHOS), and autophagy—are major drivers of chemoresistance; however, the molecular mechanisms underpinning this metabolic reprogramming remain poorly understood. Carboxylesterase 1 (CES1), an NF-κB-regulated neutral lipase, supports malignant cell survival under nutrient-depleted conditions in other cancers, but its role in HGSOC has not been explored.

**Methods:**

We investigated the role of CES1 in i) epithelial ovarian cancer (EOC) cell lines across different histological subtypes, ii) primary HGSOC cells, iii) syngeneic and platinum-resistant HGSOC mouse models. Glucose and serum limitation was used to model metabolic stress associated with platinum resistance. CES1 activity was blocked both genetically and pharmacologically using the specific CES1 inhibitor GR-148672X. Metabolic profiling, survival analysis, lipid-droplet dynamics and autophagy assays were performed. Clinical significance was evaluated by analyzing The Cancer Genome Atlas (TCGA) cohort, stratified by chemotherapy response.

**Results:**

Metabolic stress induced a shift from glycolysis to OXPHOS and promoted platinum resistance in EOC cells. This adaptation was coincident with an upregulation of CES1, which supported lipolysis, fatty acid oxidation, and autophagy/lipophagy. CES1 inhibition disrupted these processes leading to apoptosis/ferroptosis in platinum-resistant EOC lines. Oleate supplementation effectively reversed CES1-mediated biological effects, confirming their dependence on CES1-driven lipid mobilization. CES1 blockade also killed primary HGSOC cells with oxidative dependency for survival, impaired tumor growth and metastatic dissemination in *Trp53*^−/−^ ID8-Luc allografts and retained robust antitumor activity in platinum-resistant HGSOC xenografts. Further, in the TCGA dataset, an elevated CES1/Carnitine Palmitoyl-transferase 1A (CPT1A) gene signature correlated with shorter overall survival (OS) and disease-specific survival (DSS) in chemoresistant, but not chemosensitive patients with HGSOC, underscoring the clinical significance of this metabolic axis in platinum resistance.

**Conclusions:**

CES1 is a key checkpoint governing metabolic plasticity in EOC including HGSOC, by linking lipid catabolism, OXPHOS, and autophagy to chemoresistance under conditions of metabolic pressure. Targeting CES1 selectively compromises tumor cell survival under nutrient deprivation, uncovering a metabolic vulnerability that may be exploited to treat aggressive platinum-resistant HGSOC that rely on OXPHOS to thrive.

**Supplementary Information:**

The online version contains supplementary material available at https://doi.org/10.1186/s13046-026-03766-8.

## Background

Epithelial ovarian cancer (EOC) is a leading cause of cancer-related deaths in women [1]. It encompasses a highly heterogeneous group of at least five distinct tumor subtypes, each with unique histological features, origins, risk factors, clinical outcomes, and responses to treatment: high-grade serous carcinoma (HGSOC), low-grade serous carcinoma (LGSOC), endometrioid carcinoma (EOVC), clear-cell carcinoma (OCCC), and mucinous carcinoma (MOC). Among these, HGSOC is the most prevalent and lethal subtype, responsible for about 80% of all EOC fatalities [2, 3]. Despite fundamental improvements in the molecular understanding of disease biology, the mortality rate for HGSOC has remained largely unchanged over the last few decades [4]. Most patients present with advanced disease and often already have peritoneal metastases at diagnosis, which are a major contributor to the high fatality rate. Although treatment with aggressive cytoreductive surgery and neo/adjuvant platinum/taxane-based chemotherapy is generally effective, more than 80% of HGSOC cases which initially respond to this treatment eventually relapse and develop chemoresistance [5, 6]. The remaining 15% of HGSOCs are resistant to platinum-based therapy from the outset and exhibit no response to treatment and disease progression despite chemotherapy [7]. Among non­HGSOC subtypes, LGSOC, OCCC and MOC are particularly resistant to standard chemotherapy regimens [8]. Except for anti-angiogenic therapy and poly (ADP-ribose) polymerase (PARP) 1 inhibitors, which are effective in a minority of HGSOC patients, there is no successful targeted therapy in treating metastatic HGSOC, partly due to the lack of druggable oncogenic mutations. Thus, managing patients with resistant/refractory HGSOC remains a major clinical problem urgently requiring mechanistically novel treatment strategies.

Tumor cells must metabolically adapt to the nutrient- and oxygen-depleted conditions of the primary and metastatic tumour microenvironment (TME) in order to survive, proliferate, and colonize ectopic sites [9]. Under metabolic stress resulting from the low glucose and glutamine concentrations normally present in TME, cancer cells utilize alternative sources of energy, including endogenous lipids to fuel FAO and other cellular nutrients derived from autophagy [10]. Notably, these processes have also been implicated in promoting chemoresistance, thus underscoring the central role that free fatty acids (FFA) and energy metabolism play in tumour progression and therapy resistance [11]. Although the underlying mechanisms are not yet fully defined, a shift toward more energy-demanding metabolic pathways appears to be required to sustain the expression and the activity of multidrug resistance (MDR) transporters, which rely on ATP to mediate drug efflux [12]. Recent studies utilizing multi-omic have provided deeper insights into the metabolic dependencies that characterize platinum-refractory HGSOC. Huang et al*.* have reported that a metabolic reprogramming toward FAO enables HGSOC cells to survive chemotherapy through enhanced lipid utilization. The same study identified CPT1A, the rate-limiting enzyme in FAO, as a potential therapeutic target in platinum-resistant tumors [13]. Further, a comprehensive proteogenomic analysis of chemorefractory HGSOC demonstrated that a series of coordinated metabolic, proteomic, and genomic alterations converge on promoting tumor persistence under selective pressure from chemotherapy [13]. This study demonstrated that the dysregulation of cellular pathways, such as OXPHOS and NF-κB signaling, plays a key role in tumor cell survival and disease progression during platinum-based chemotherapy [13]. These findings establish a direct link between metabolic plasticity, lipid metabolism, and platinum-based therapy resistance, thereby exposing a metabolic vulnerability that can be targeted to effectively treat advanced, chemorefractory HGSOC.

CES1 is an NF-κB-regulated triacylglycerol (TAG) and cholesteryl ester (CE) lipase that plays an essential role in cancer cell survival and metabolic adaptation to nutrient-depleted microenvironments in aggressive colorectal carcinoma (CRC) and other human cancers [14]. Mechanistically, CES1 mediates two essential cytoprotective functions in CRC cells: *(1)* It enhances the breakdown of TAGs and CEs stored in lipid droplets (LD) to mobilize FFAs to fuel FAO and OXPHOS; *(2)* it prevents the toxic accumulation of neutral lipids that leads to reactive oxygen species (ROS) formation and phospholipid peroxidation, which trigger apoptosis and ferroptosis, respectively [14]. Interestingly, a previous study by Li et al*.* [15] demonstrated a pro-survival role for CES1 also in hepatocellular carcinoma (HCC), where the disruption of lipid metabolism by targeting the CES1-PPARα/γ-SCD axis was shown to sensitize HCC cells to cisplatin chemotherapy.

We investigated whether CES1 promotes metabolic adaptation and cancer-cell survival in platinum-resistant EOC, given the reliance of these tumors on lipid metabolism and aberrant NF-κB activation and their propensity to metastasize adipocyte-rich microenvironments in the peritoneal cavity and omentum. We show that metabolic stress induces platinum chemoresistance and CES1 is essential in this model for supporting EOC cell survival under conditions of nutrient limitation via a mechanism that enhances metabolic adaptation and autophagy. We find that CES1 inhibition counteracts tumor growth in vivo*,* including in a platinum-resistant HGSOC xenograft model, and effectively kills platinum-resistant EOC cell lines and primary human HGSOC cells, relying on OXPHOS for survival, via apoptosis, ferroptosis and other cell death pathways. These findings identify CES1 as an actionable metabolic vulnerability in chemoresistant EOC and support CES1 inhibition as a promising therapeutic strategy for platinum-resistant disease. Accordingly, we find that an elevated *CES1/CPT1A* signature portends a worse prognosis in patients with platinum-refractory, but not platinum-sensitive, HGSOC, underscoring the clinical significance of the CES1-dependent reprogramming toward oxidative metabolism in chemoresistant human tumors.

## Methods

### Cell culture and lentiviral infection

The human EOC cell lines, CAOV3, KURAMOCHI, OVSAHO, OVKATE, OVTOKO, SKOV3, EFO-21, OV-56, OVCA-429, and ES-2 were purchased from CliniSciences and cultured in accordance with manufacturer’s specifications. CAOV3, SKOV3, EFO-21 and ES-2 were cultured in DMEM (Dulbecco’s Modified Eagle’s Medium) supplemented with 10% Fetal Bovine Serum (FBS; GIBCO); KURAMOCHI, OVSAHO, OVKATE, OVTOKO, OV-56, and OVCA-429 were cultured in RPMI 1640 (Roswell Park Memorial Institute) supplemented with 10% FBS. All media were further supplemented with 2 mM L-glutamine (Euroclone), 150 U/mL penicillin and 200 U/mL streptomycin (Euroclone).

Cells were cultured in a humidified incubator at 37 °C with 5% CO₂. All cell lines were routinely tested for mycoplasma contamination using the Mycoplasma Detection Kit (G238, ABM). Cell line authentication (STR) was carried out for all EOC cell lines. For experiments under energy stress, EOC cells were cultured in glucose-free DMEM or RPMI (Gibco) supplemented with cell line-specific concentrations of FBS and D-glucose (G8769, Sigma-Aldrich), as detailed in the following table:

**Table:** 

Cell line	Medium without glucose	FBS	D-glucose
CAOV3	DMEM	2%	1 mM
KURAMOCHI	RPMI-1640	2%	1 mM
OVSAHO	RPMI-1640	2%	1 mM
OVKATE	RPMI-1640	2%	1 mM
OVTOKO	RPMI-1640	5%	1 mM
SKOV3	DMEM	5%	1 mM
EFO-21	DMEM	2%	1 mM
OV-56	RPMI-1640	5%	1 mM
OVCA-429	RPMI-1640	5%	1 mM
ES-2	DMEM	5%	0,5 mM

ID8 *Trp53*^−/−^ [16]—kindly provided by Prof. Ian McNeish (imperial College London)—were engineered to stably express firefly luciferase (Luc) at Mario Negri Institute for Pharmacological Research (IRFMN) and maintained in high-glucose DMEM medium supplemented with 2 mM glutamine, 150 U/mL penicillin, 200 U/mL streptomycin, 4% FBS, ITS Liquid Media Supplement (5 µg/mL insulin, 5 µg/mL transferrin and 5 ng/mL sodium selenite) (Gibco), and 1 µg/mL blasticidin (Gibco) at 37 °C with 5% CO2.

KURAMOCHI-Luc were kindly provided by Prof. Saula Checquolo (La Sapienza University of Rome) and maintained in RPMI medium supplemented with 2 mM glutamine, 200 U/mL streptomycin and 10% FBS at 37 °C with 5% CO_2_.

The production of high-titre lentivirus in HEK293T cells was carried out using Lipofectamine 3000 Transfection Reagent (#L3000015, Thermo Fisher) in accordance with the manufacturer’s specifications. Lentiviral infections were carried out as previously described [17]. The DNA sequences of the shRNAs used for targeting the human *CES1* and *RELA* genes and the non-specific (ns) controls in the pLKO.1 or pLentiLox3.7 lentiviral vector were previously described and validated for specificity [14]. Likewise, the TWEEN lentiviral vector used for *Ces1d* overexpression was previously described [14].

The following reagents were used for cell treatments: GR-148672X (10 μM) from Chemgood LLC, Tween 80 (2%, #P8192) from Sigma Aldrich, Oleic acid (50 μM, #S-O1383) from Merck, Carboplatin (10–100 μM, #C2538) from Sigma Aldrich, Bafilomycin A1 (50 nM, #B1793) from Sigma Aldrich, DGAT1 inhibitor (40 μM, #A1737 [18]) from Sigma Aldrich, Triacsin C (10 μM, #10,007,448–1 [14]) from Cayman Chemical.

### Cell viability assay

Cell viability assay was carried out using CellTiter-Glo® 2.0 Cell Viability Assay reagent (G9242, Promega) according to the manufacturer''s protocol. Cell counting was performed using Cyto Smart cell counter (Corning). At the endpoint, cells were lysed with CellTiter-Glo® 2.0 reagent and luminescence was determined with Infinite® 200 PRO, TECAN, Männendorf, Switzerland.

### Activity-based protein profiling (ABPP) and LC–MS/MS

HepG2 and EFO-21 cell pellets were resuspended in 50 mM Tris–HCl buffer (pH 7.5) and subjected to mechanical disruption using a gentleMACS™ Dissociator to ensure efficient homogenization. After centrifugation of cell lysates, total protein concentration was determined using Bradford protein assay. Protein aliquots (230 µg each) were divided into the following groups and processed as follows: *i)* no-probe group [lysates processed without probe incubation]; *ii)* control group [lysates incubated with DMSO for 1 h at room temperature, followed by incubation with ActivX® Desthiobiotin-FP probe (#88,317, Thermo Fisher Scientific) for 1 h on the rotator]; *iii)* GR-148672X group [lysates pre-treated with GR-148672X, 50 µM, for 1 h at room temperature, followed by incubation with ActivX® probe for 1 h on the rotator]. Probe-labeled proteins were purified by biotin-affinity pull-down using streptavidin/agarose-coated beads, according to the manufacturer’s protocol. Bound proteins were subjected to sequential washes with 5 M urea, 0.1% Triton X-100, and PBS 1X. Following affinity purification, samples were processed according to the manufacturer’s instructions (ActivX® Serine Hydrolase Probes Kit, Cat. No. 88317, Thermo Fisher Scientific) following the Active-site Peptide Enrichment workflow. Eluted proteins were digested, and enriched active-site peptides were analysed by LC–MS/MS. Data were obtained from two independent experiments. The annotated serine hydrolases were from Cravatt et al*.* [19, 20] and NIH Molecular Libraries Program (NCBI database).

### Oxygen consumption rate (OCR) analysis

For bioenergetic profiling, EOC cells were seeded onto Seahorse 96-well plates. Mitostress, ATP rate and Mito fuel stress tests were conducted using a XF-96 Extracellular Flux Analyzer (Agilent Seahorse), and oxygen consumption rates (OCR) was measured in XF Assay Medium (#102,365, Agilent Seahorse) containing 1 mM pyruvate, 2 mM glutamine, and 10 mM glucose. In the Mitostress test, OCR was measured following injection of 10 μM GR-148672X or vehicle (DMSO) in combination with 50 mM 2-deoxy-D-glucose (#D8375, Sigma Aldrich); 1,5 μM oligomycin A (#75,351, Sigma Aldrich); 75 μM 2,4-Dinitrophenol (2,4-DNP; #D198501, Sigma Aldrich); 0,5 μM rotenone (#R8875, Sigma Aldrich); and 0.5 μM antimycin A (#A8674, Sigma Aldrich). ATP production and proton leak were calculated in accordance to the manufacturer’s specifications as follows: ATP production-coupled respiration (*i.e*., the last measurement before oligomycin A injection after subtracting the minimum rate measurement after oligomycin A injection), and proton leak (*i.e.*, the minimum rate measurement after oligomycin A injection after subtracting non-mitochondrial respiration).

ATP production rates were measured using the XF Real-Time ATP Rate Assay Kit (103,592–100, Agilent Seahorse) following the injection of GR-148672X where indicated. The detected mitochondrial OCR and glycolytic Extra-Cellular Acidification Rates (ECAR) were then transformed into mitochondrial (mito-ATP) and glycolytic (glyco-ATP) ATP production rates, using validated algorithms implemented in the Agilent Seahorse software and expressed as percentages. The metabolic dependency of cells for glutamine and long chain fatty acids (FAO) was measured by using XF Mito Fuel Flex Test Kit (103,260–100, Agilent Seahorse), which detects the basal state mitochondrial fuel oxidation in live cells by injecting BPTES, Etomoxir, and UK5099 according to the manufacturer’s specification. All values were normalized on a per-cell basis.

### RNA Extraction and Quantitative Real-Time Polymerase-Chain Reaction (Q-PCR) analysis

Total RNA was extracted using Quick-RNA™ MiniPrep kit (Zymo Research) according to the manufacturer’s specifications. The isolated RNA was spectrophotometrically quantified and 1 μg of RNA was reverse transcribed using the High-Capacity cDNA Reverse Transcription kit (Applied Biosystems). q-PCRs were carried out on an ABI 7500 Fast real-time PCR machine (Life Technologies) using Taq Man Universal Master Mix and the following predesigned TaqMan® Gene Expression Assays: Actin Human Hs01060665_g1 FAM, CES1 Human Hs00275607_m1 FAM, Ces1d Mm00474816_m1 FAM. Ct values were normalized to Actin gene expression.

### Western blot analysis

Total cell extracts were prepared in Lysis buffer (50 mM Tris–HCl (pH 7.4), 150 mM NaCl, 1% Triton X-100, 1 mM EDTA, 1 mM Na_3_VO_4_, 50 mM NaF) containing complete mini EDTA-free protease inhibitors (Roche) and protein concentration was assessed by the BCA assay kit (Thermo Fisher). Primary antibodies used were as follows: CES1 (ab45957 Abcam), LC3B (D11) XP (3868 Cell Signaling), caspase 3 (9662 Cell Signaling), cleaved caspase 3 (Asp175) (5A1E) (9664 Cell Signaling), PARP1 (9542 Cell Signaling), GPX4 (ab125066 Abcam), RELA (8242 Cell Signaling), CPT1A (ab128568 Abcam), LC3B (83,506 Cell Signaling), Actin (C-11) (sc-1615 Santa Cruz Biotechnology). Western blot detection was performed using ECL (Amersham).

### Flow cytometry

For CES1 intracellular staining, 1 × 10^6^ EOC cells were fixed and then permeabilized using the following commercial buffers: Phosflow Fix Buffer I and Perm/Wash Buffer, (BD Biosciences). Permeabilized cells were stained with anti-CES1 (ab68190, Abcam) and AlexaFluor 488 anti-rabbit (Immunological Sciences). Cyto-ID® Autophagy Detection Kit 2.0 (ENZ-KIT175-0050 Enzo Life Sciences) was used for the selective quantification of accumulated autophagic vacuoles according to the manufacturer’s specification. Nucleosomal DNA fragmentation was assessed by using propidium iodide (PI, #550,825, BD Biosciences) nuclear staining assays and processed according to the manufacturer’s specification. For the detection of lipid peroxidation, ES-2 cells were loaded with 10 μM of C11-BODIPY 581/591 C11 (D3861, Thermo Fisher Scientific) for 3 h at 37 °C and then processed according to the manufacturer’s specification. Data were acquired using a FACSCanto II flow cytometer (BD, Biosciences) and analyzed using FlowJo software.

### Fluorescence confocal microscopy

BODIPY™ 493/503 (#D3922, Thermofisher) was used to stain neutral lipids at the final concentration of 3 mM. LysoTracker™ Red DND-99 (#L7528, ThermoFisher) was used to stain lysosomes at the final concentration of 50 nM. Cells were incubated with the dyes for 30 min and then slides were mounted. Nuclei were stained using Anti Fade™ Mounting Medium with DAPI (Immunological Sciences). Confocal images were acquired with a Leica Stellaris 8 TauSTED microscope (Leica Microsystems) equipped with a White Light Laser (WLL) using an oil-immersion objective HC PL APO 63x/1.40 NA. Images were processed with LAS X software (version 4.7.0.28176). Colocalization was analysed through the semi-automated Fiji plugin “JaCoP” to calculate the Pearson’s correlation coefficient (PCC). Lipid droplet counts were performed using Image J software.

### Holotomography-based LD imaging

Label-free images were acquired using a 3D-Cell Explorer-fluo (Nanolive SA, Tolochenaz, Switzerland) microscope, as previously described [21]. Briefly 2 × 10^5^ KURAMOCHI cells were plated in a 35 mm Ibidi polymer coverslip bottom dish. After 48 h, cells were placed in the incubator chamber of the microscope and imaged. Images were reconstructed as 3D volumes by the EVE software, controlling the 3D cell explorer microscope and data management. Analysis of LD metrics has been performed using the Nanolive software Smart Lipid Droplets Assay.

### Primary HGSOC cells

Primary cell cultures were established from peritoneal ascites and omental metastasis of chemo-naïve HGSOC patients operated at the European Institute of Oncology (IEO, Milan). Tumor samples were collected at the moment of primary debulking surgery. Human samples were collected in accordance with the Declaration of Helsinki, and the study was approved by the Ethics Committee for Clinical Research of the Istituto Europeo di Oncologia (Approval number R789-IEO). All participants provided written informed consent before their inclusion in the study. The purity of primary cell culture was consistently over 95%. Tissue isolation and culture conditions of primary cells were performed as described previously by Francavilla et al*.* [22]. Clinicopathological features of the patients included in this study are provided in the table below:

**Table:** 

Sample	Diagnosis	Grade	FIGO stage	Age	Chemotherapy status
P1	HGSOC	3	IIIC	45	Naïve
P2	HGSOC	3	IV	73	Naïve
P3	HGSOC	3	IIIC	64	Naïve

### Animal studies

C57BL/6 female mice were purchased from Inotiv. All animal experiments were approved by the Institutional Ethics Committee for Animal Use (OPBA) and by the Italian Ministry of Health (authorization number 1038/2023-PR) and were performed following to the 3Rs’ recommendations (Reduction, Refinement and Replacement). All experimental procedures were conducted in accordance with ethical guidelines and approved by the appropriate institutional review board. Measures were taken to minimize pain, suffering, and distress, including the use of anaesthesia and analgesia where applicable, as well as the implementation of humane endpoints and routine monitoring for signs of discomfort. Animals were monitored for clinical signs including weight loss exceeding 20%, lethargy, abnormal posture or gait, decreased food or water intake, and signs of infection or inflammation. Mice were housed in ventilated cages with free access to autoclaved food and water. They were kept on autoclaved bedding in the Conventional Mouse Facility at the University of L’Aquila under a 12-h light/dark cycle. For the syngeneic model of HGSOC, 8-week-old C57BL/6 female mice (Inotiv) were intraperitoneally inoculated with 1 × 10^6^*Trp53*^−/−^ ID8-Luc cells. The inoculated ID8-Luc *Trp53*^−/−^ cells were resuspended in 200 µL of DMEM high glucose and saline solution (1:5) and tumours were allowed to grow as previously done by Verzella et al*.* [23]. Fifteen days after the injection of the cells, mice were randomized into treatment groups and then treated intraperitoneally with GR-148672X (50 mg/kg of body weight; *n* = 8) or vehicle (*n* = 5), twice a day. For in vivo administration, GR-148672X was dissolved in DMSO at the final concentration of 100 mM and then diluted in 2% Tween 80/saline solution to a stock concentration of 5 mg/mL. For the xenograft model of HGSOC, 6-week-old NOD.CB17‐ Prkdc scid IL2rg tm1/Bcgen female mice (Inotiv) were intraperitoneally inoculated with 5 × 10^6^ KURAMOCHI-Luc cells resuspended in 200 µL of RPMI high glucose and saline solution (1:5) and tumours were allowed to grow as previously reported [24]. Fifteen days after cell injection, mice were randomized and treated intraperitoneally with vehicle (n = 4), GR-148672X (50 mg/kg body weight, twice daily; n = 5), carboplatin (20 mg/kg body weight, every other day; n = 4), or the combination of both agents (n = 5). At the experimental endpoint, mice were humanely euthanized in accordance with institutional animal care guidelines and all visible tumor deposits were recorded by photography.

### In vivo bioimaging

For bioimaging, mice were intraperitoneally injected with 20 µg/g of body weight of D-luciferin (Invitrogen). After ten minutes of incubation, mice were anesthetised by using 2% isoflurane with the oxygen flow rate set at approximately 2.0 L/min. Then, bioluminescence from ID8-Luc *Trp53*^−/−^ or KURAMOCHI-Luc tumours was acquired in vivo using a IVIS Spectrum imaging system (PerkinElmer) and imaging data were analysed using LivingImage software.

### Bioinformatic data

The UCSC Toil RNA-Seq Recompute data [25] from *The Cancer Genome Atlas* (TCGA) Ovarian (OV) program were used to obtain gene expression levels and were downloaded via the UCSC Xena browser. Disease-Specific Survival (DSS), Overall Survival (OS), and clinical data were derived from the TCGA Pan-Cancer Atlas publication by Liu et al*.* [26] and downloaded from the Genomic Data Commons repository (https://gdc.cancer.gov/node/905/). Time to Treatment Failure (TTF) and chemotherapy outcomes for patients with HGSOC from TCGA were obtained from the study by Villalobos et al*.* [27]. The following criteria were used: If TTF was classified as “Free” and Days-Off-Platinum was < 30.5 days (corresponding to 1 month), patients were defined “refractory”; if TTF was classified as “Free” and Days-Off-Platinum was between 30.5 and 183 days (1–6 months), patients were defined “resistant"; and “sensitive” was used if Days-Off-Platinum was > 183 days (more than 6 months). For DSS and OS analyses, patients with both specific survival value (DSS or OS) and TTF data available were included. A maximum follow-up period of 7 years was considered. Patients were stratified into two groups based on the mRNA expression levels for the single gene (*CES1* or *CPT1A*) and the average of the Z-score for the gene signature and using the first tertile as threshold. Patients with gene or signature expression levels above the first tertile were classified as “high expression”, while those with levels below the first tertile were classified as “low expression”. Survival curves were estimated using the Kaplan–Meier method, and statistical differences were tested using the logrank test. p values < 0.05 were statistically significant.

Global proteomic data were obtained from the study by Chowdhury et al*.* [28] and batch-corrected global proteomic data from the FFPE discovery cohort, prior to imputation, were used for the analyses. The autophagy signature was derived from the Reactome database (version 82) and downloaded using PathwayBrowser (version 3.6). Protein expression values were scaled according to the Z-scores, and the average of these values was used as the signature score.

All analyses were performed using RStudio software (R version 4.5.1).

### Statistical analyses

For the analysis of biological data, the statistical significance for two-sample comparisons was calculated using two-tailed Student’s t-test. The statistical significance for multiple-sample comparisons was calculated using one-way ANOVA. Differences were considered to be statistically significant if the *p* value, after correction in the case of multiple tests, was lower than 0.05. Experiments were repeated three times unless specified otherwise.

## Results

### Energy stress promotes the metabolic reprogramming of EOC cells toward OXPHOS and enhances resistance to carboplatin

**Fig. 1:**
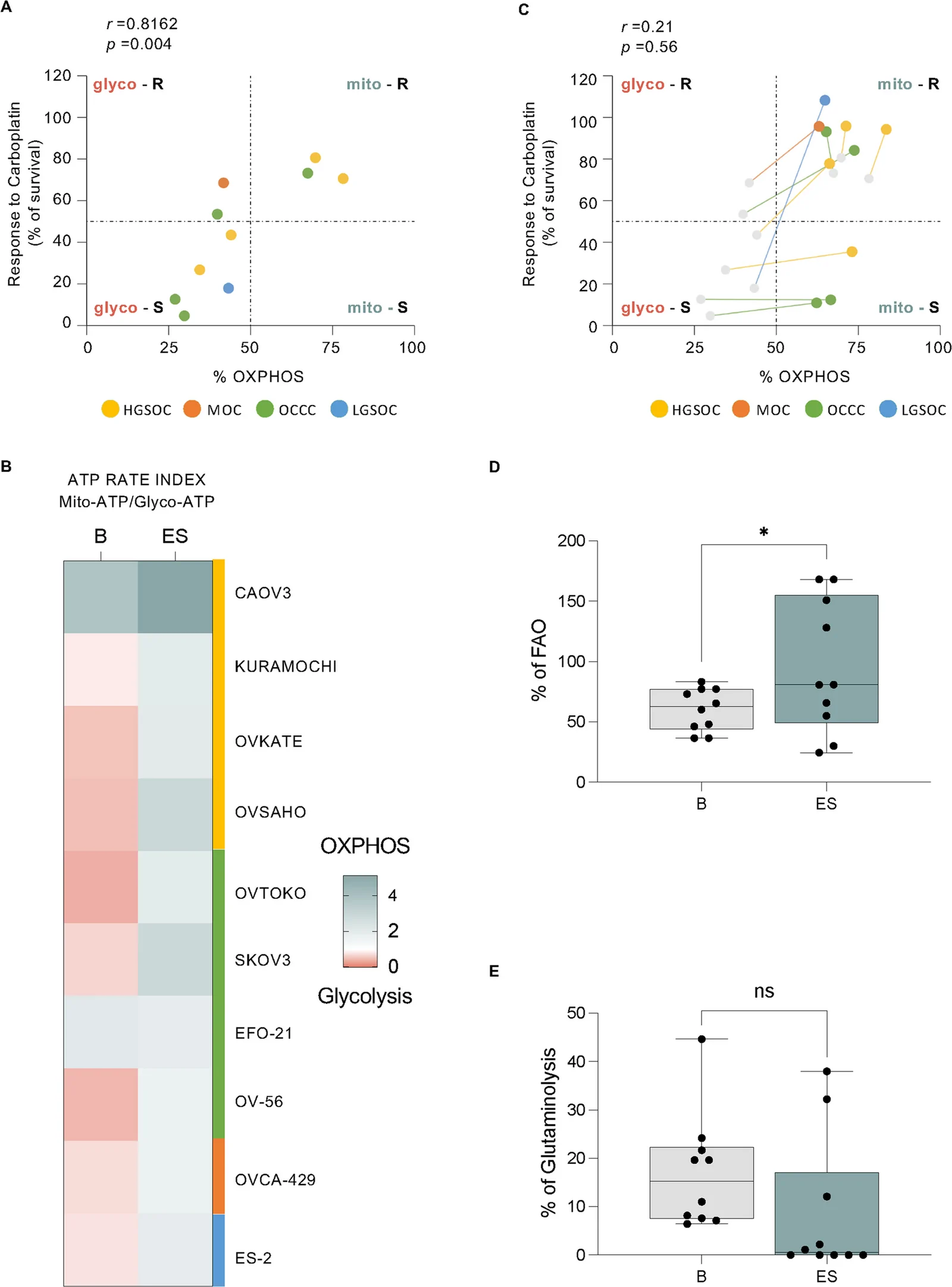
Energy stress-induced metabolic shift to OXPHOS supports carboplatin resistance in EOC cells**. A** Correlation between response to carboplatin treatment and percentage of OXPHOS used by EOC cells under basal conditions (standard glucose and serum concentrations). Cell survival was measured by CellTiter-Glo® 2.0 assay after a 3-day treatment with 100 μM carboplatin. The percentage of OXPHOS contribution for each cell line was calculated from the mitochondrial ATP (mitoATP) values obtained with the Seahorse XFe96 ATP rate assay. **B** Heatmap showing the ATP rate index in the indicated EOC cell lines cultured under basal (B) or energy stress (ES, glucose and serum limitation) conditions for 3 days. **C** Correlation between response to carboplatin treatment and percentage of OXPHOS used by EOC cells under ES condition and calculated as in (A). **D**-**E** Box plots showing the percentage of fatty acid oxidation (FAO) (D) and glutaminolysis (E) dependency in EOC lines under B or ES, as indicated, measured by using XF Mito Fuel Flex Test Kit (Seahorse XFe96). Shown in the box plots are the median values (horizontal lines), 25th to 75th percentiles (box outlines), and highest and lowest values within 1.5 times the interquartile range (vertical lines). Notches denote the 95% confidence interval of the medians. Values are shown as mean ± SEM. Statistical significance was calculated by 2-tailed Student’s *t* test. *, *p* < 0.05

Lipid metabolism promotes tumour progression in the nutrient-depleted TME where glucose and other energy sources are scarce [29]. Recent studies have shown that OXPHOS and FAO are increased in platinum-resistant EOC cell lines and primary HGSOC cells from patients [13, 30]. Consistent with these findings, our analysis of a panel of cell lines encompassing different histopathological types of EOC [31] – including HGSOC (CAOV3, KURAMOCHI, OVSAHO, OVKATE), OCCC (OVTOKO, EFO-21, OV-56, SKOV3), LGSOC (ES-2), and MOC (OVCA-429) – demonstrated a strong correlation between the OXPHOS usage for energy provision and sensitivity to carboplatin, with more glycolytic EOC cell lines being more sensitive to carboplatin and more oxidative cell lines being more resistant to this agent (Fig. 1A, S1A-S1B). Next, to mimic conditions of nutrient deprivation, which occur during the shedding of cancer cells into the ascitic fluid and metastatic spread to the peritoneum, we cultured EOC lines under glucose and serum limitation, hereafter referred to here as energy stress conditions. Notably, under energy stress, EOC cells that relied on glycolysis under basal conditions reprogrammed their metabolism toward OXPHOS (Fig. 1B, S1C) and the majority of them developed resistance to carboplatin (Fig. 1C). In agreement with previous studies, this suggests that the energy stress-induced metabolic rewiring toward OXPHOS induces carboplatin chemoresistance. This reprogramming of EOC cells to OXPHOS was largely driven by FAO, rather than amino acid metabolism, as FAO flux was increased, whereas glutaminolysis was reduced in most EOC cell lines cultured under ES (Fig. 1D-1E). Collectively, these data indicate that an increased reliance on FAO promotes cancer cell survival and contributes to chemotherapy resistance under conditions of nutrient paucity.

### Carboxylesterase 1 (CES1) is upregulated by energy stress and promotes metabolic adaptation and cell survival in EOC

**Fig. 2:**
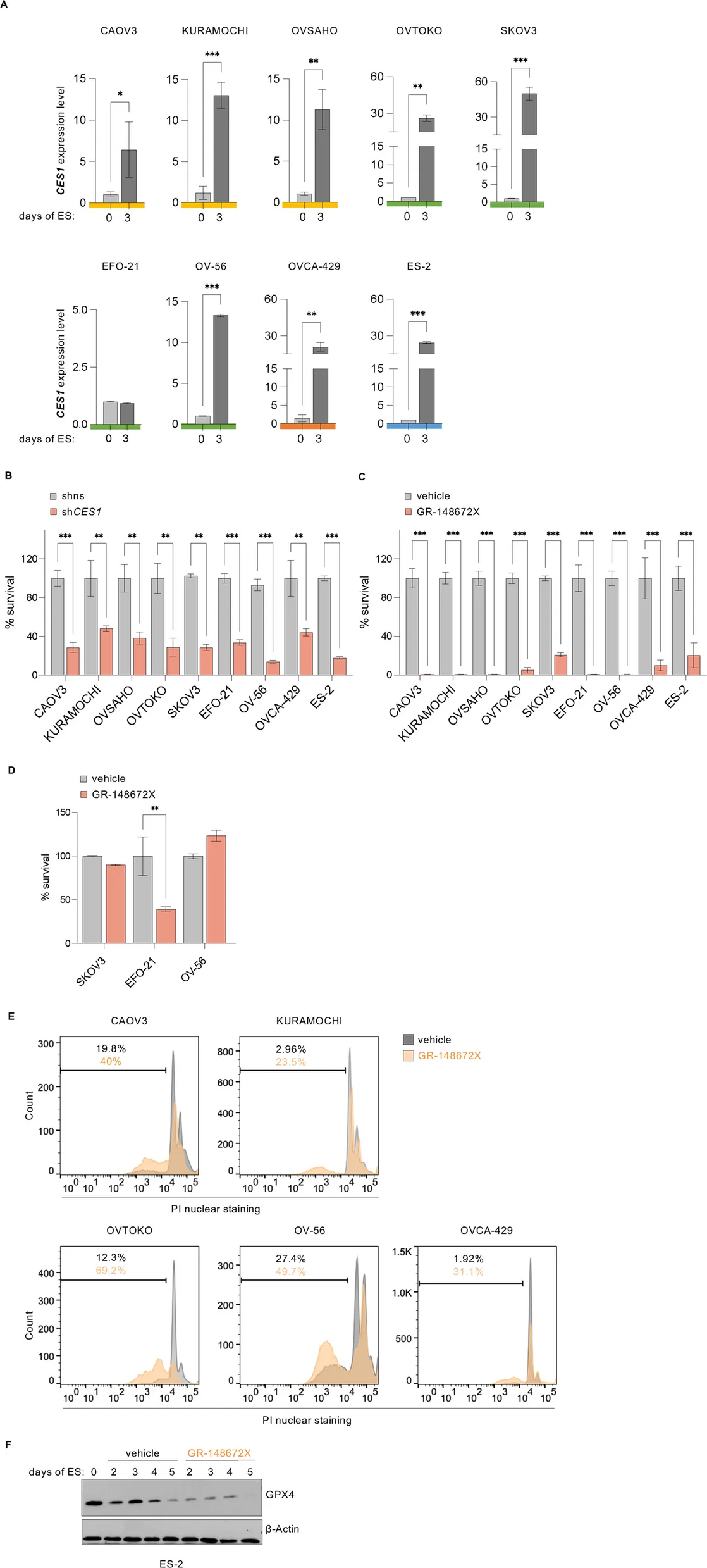
*CES1* is induced during nutrient stress and sustains EOC viability. **A** qRT-PCR showing *CES1* mRNA levels in the indicated EOC cell lines cultured under basal condition (0) or for 3 days under energy stress (ES). **B** CellTiter-Glo® 2.0 viability analysis of EOC cells expressing *CES1*-specific (sh*CES1*) and nonspecific (ns) shRNAs after a 4-day under ES. **C** CellTiter-Glo® 2.0 viability analysis of EOC cells after a 4-day treatment with GR-148672X or vehicle under ES. **D** CellTiter-Glo® 2.0 viability analysis of SKOV3, EFO-21 and OV-56 cells after a 4-day treatment with GR-148672X or vehicle under basal conditions. **E** Propidium iodide staining showing apoptotic DNA fragmentation in EOC cells treated as in B after 3 days of ES. **F** Western blots showing the protein levels of ferroptotic (GPX4) marker in ES-2 cells treated as in B. β-actin is shown as loading control. **A**-**D** Values are shown as mean ± SD. Statistical significance was calculated by 2-tailed Student’s *t* test. *, *p* < 0.05, **, *p* < 0.01, ***, *p* < 0.001

CES1 has been shown to promote metabolic adaptation in cancer cells, and its pharmacologic blockade has resulted in a profound inhibition of CRC growth and sensitization of HCC to platinum-based chemotherapy in animal models, in vivo [14, 15]. Thus, we hypothesised that CES1 could contribute to the metabolic reprogramming of EOC cells toward OXPHOS during chemoresistance-inducing energy stress. To test this hypothesis, we evaluated *CES1* mRNA and protein expression in EOC cells under basal conditions and upon energy stress (Fig. 2A, S2A-2B). While *CES1* was constitutively expressed at relatively high levels in only three EOC cell lines (*i.e.*, SKOV3, OV-56, EFO-21) (Figure S2A-S2B), all cell lines upregulated *CES1* under ES, except for the EFO-21 cell line, which exhibited elevated *CES1* expression under both basal and energy stress conditions (Fig. 2A, S2C).

To explore potential mechanisms regulating *CES1* expression in EOC cells, we performed gene silencing experiments targeting NF-κB/*RELA*, a transcription factor previously implicated in CES1 transcriptional control [14]. *RELA *silencing resulted in a clear reduction of *CES1* levels (Figure S2D), supporting its role in *CES1 *regulation also in EOC cells.

Further, *CES1* knockdown or pharmacologic blockade with the specific inhibitor GR-148672X led to cell death in all platinum-resistant EOC lines under energy stress (Fig. 2B-2C, S2E). Thus, irrespective of its basal expression levels, CES1 is essential for metabolic adaptation to energy stress across all EOC cell lines tested. Consistent with the multifactorial nature of the adaptive metabolic rewiring induced by energetic stress that promotes chemoresistance, CES1 overexpression alone did not significantly alter the response to carboplatin (Figure S2F-S2G).

To further support the pharmacological targeting of CES1, we next evaluated the selectivity of GR-148672X. A global activity-based protein profiling (ABPP) of fluorophosphonate (FP) probe-labeled serine hydrolases by LC–MS analysis in HepG2 HCC cells confirmed the on-target specificity of GR-148672X for CES1, with no off-target activity versus the paralog, CES2, or the majority of the other serine-hydrolase superfamily members expressed in HepG2 cells (Figure S2H). Consistent with the results obtained in hepatocellular cells, GR-148672X selectively inhibited CES1 activity in EOC cells with minimal effects on other detectable serine hydrolases (Fig. S2I). Interestingly, in EFO-21 cells – which exhibit high basal CES1 expression and OXPHOS-dependent ATP production exceeding 65% – CES1 inhibition also induced cell death under basal conditions (Fig. 2D, S1A). This suggests that the outcome of CES1 blockade has contextual specificity for cellular conditions of OXPHOS dependency, which may occur in the TME.

Notably, the type of cell death induced by CES1 inhibition varied considerably across cell lines. For example, in most cell lines we predominantly observed the induction of apoptosis, as shown by an increase in nucleosomal DNA fragmentation and cleavage of procaspase-3 and PARP1 (Fig. 2E, S2J), whereas in others (*e.g.*, ES-2 cells) cell death appeared to largely occur via ferroptosis, as shown by a reduction in glutathione peroxidase 4 (GPX4) protein levels and the increase of lipid peroxidation (Fig. 2F, S2K).

These results align with those from previous studies which reported the activation of both apoptosis and ferroptosis upon CES1 inhibition, under starvation or in combination with cisplatin, in CRC and HCC cells, respectively [14, 15].

To further evaluate the therapeutic potential of CES1 inhibition in a platinum-resistant setting, we assessed the effect of GR-148672X in combination with carboplatin in KURAMOCHI cells cultured under energy stress previously shown to enhance carboplatin resistance. Under these conditions, carboplatin treatment failed to reduce cell viability and was associated with increased survival compared with vehicle-treated controls (Figure S2L). In contrast, GR-148672X significantly reduced cell viability. The combination of GR-148672X and carboplatin did not provide additional benefit compared with GR-148672X monotherapy, highlighting the strong efficacy of CES1 inhibition as a single agent in this setting (Figure S2L). These findings are likely attributable to the strong single-agent activity of GR-148672X observed in vitro. Collectively, these results identify CES1 as a druggable therapeutic target for overcoming energy stress-induced chemotherapy resistance in advanced EOC.

### CES1 blockade impairs lipolysis-driven OXPHOS and autophagy

**Fig. 3:**
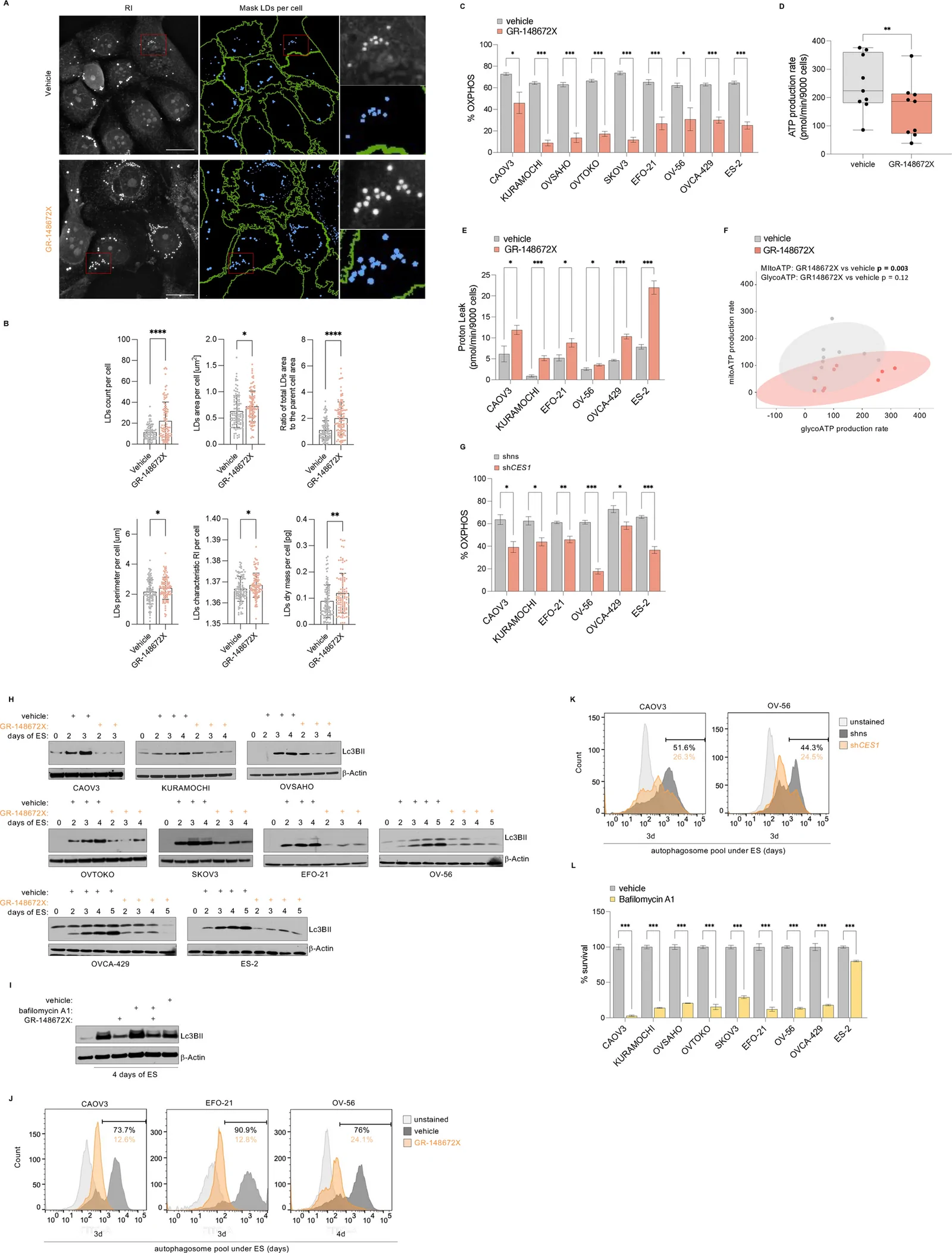
Blocking CES1 impairs lipid-fueled OXPHOS and autophagy. **A** Representative images generated by 3D holotomographic Nanolive microscope showing LDs in KURAMOCHI cells after 2 days of treatment with GR-148672X or vehicle under energy stress. Scale bar: 20 µm. **B** Quantitative analysis of LD metrics by Smart Lipid Droplets Assay in cells from A (*n* = 106–110 cells per condition from three independent biological replicates). **C** Percentage of OXPHOS usage in EOC cells treated with GR-148672X or vehicle after 3 days of energy stress as calculated in Fig. 1A. **D** Box plots showing ATP production rate in cell lines from C in presence of GR-148672X or vehicle. Shown in the box plots are the median values (horizontal lines), 25th to 75th percentiles (box outlines), and highest and lowest values within 1.5 times the interquartile range (vertical lines). Notches denote the 95% confidence interval of the medians. **E** Assessment of proton leak as a mitochondrial respiratory parameter in cells from C in presence of GR-148672X or vehicle. **F** Distribution of mitoATP and glycoATP production across cell lines from C. The 95% confidence ellipses for both the vehicle and treated groups are shown. A Student''s t-test on the mitoATP and glycoATP production rate was performed, and a statistically significant difference was found (p = 0.003) for mitoATP. **G** Percentage of OXPHOS after 3 days of energy stress in EOC cells treated with shns or sh*CES1 *calculated following the ATP rate test by Seahorse XFe96. **H** Western blots showing the protein levels of LC3BII in EOC cells treated with GR-148672X or vehicle at the indicated time points under energy stress (ES). β-actin is shown as loading control. **I** LC3BII expression levels of OV-56 cells treated as indicated after 4 days of energy stress. β-actin is shown as loading control. **J**-**K** Cyto-ID FACS analysis showing autophagosome accumulation in the indicated EOC cells treated with GR-148672X or vehicle (J) or expressing sh*CES1* or shns RNAs (K) under energy stress. **L** CellTiter-Glo® 2.0 viability analysis of EOC cells after 4 days of energy stress and treated with Bafilomycin A1 or vehicle. **B**-**G**,** L** Values are shown as mean ± SD. Statistical significance was calculated by 2-tailed Student’s *t* test. *, *p* < 0.05, **, *p* < 0.01, ***, *p* < 0.001

Recent reports indicate that CES1 regulates LD dynamics by hydrolyzing TAGs and CEs to increase FFA availability for energy production via FAO [14, 15, 32, 33]. Consistent with these reports, live imaging using label-free 3D holotomographic (HT) microscopy demonstrated a significant accumulation of LDs in EOC cells cultured under energy stress conditions and treated with GR-148672X versus vehicle-treated cells (Fig. 3A; see also Fig. 3B, top-left panel [LD counts per cells]). This increase in the number of LDs per cell was confirmed by confocal microscopy using BODIPY (Figure S3A–S3B). The analysis of HT images also revealed a significant enlargement of LDs, as shown by the increased LD area and perimeter observed in GR-148672X-treated compared to control cells (Fig. 3B, *see* top middle, top right, and bottom left panels). Further, CES1 inhibition resulted in a correspondingly higher content of neutral lipids per cell, as indicated by the increased refractive index and dry mass of LDs (Fig. 3B, *see* bottom middle and bottom right panels), in keeping with previous studies [14]. Additional parameters, such as LD eccentricity, granularity, and distance from the centroid of parent cells were not affected by CES1 blockade (Figure S3C). Notably, CES1 inhibition by GR-148672X also reduced cellular OXPHOS utilization and ATP production rate (Fig. 3C-3D). It also impaired bioenergetic parameters, including proton leak and ATP-coupled respiration (Fig. 3E, Figure S3D-S3E). Further, while it reduced mito-ATP production rate, CES1 blockade did not affect glyco-ATP production (Fig. 3F). *CES1* knockdown also reduced OXPHOS utilization (Fig. 3G), suggesting that CES1-dependent lipolysis is essential for the reprogramming EOC cells toward oxidative metabolism under energy stress conditions.

Since autophagy is a key mechanism of cell survival during nutrient deprivation and induction of therapy resistance in EOC cells [34, 35], we investigated the role of CES1 in regulating this process. As expected, we found that expression of the lipidated and active form of the autophagy marker, LC3BII, increased under energy stress [36]. LC3BII however, decreased upon CES1 inhibition, indicating that CES1 is required for autophagy induction in EOC cells cultured under energy stress conditions (Fig. 3H). Autophagy flux analysis supports this conclusion as combined treatment with GR-148672X and the autophagy inhibitor, Bafilomycin (BafA1), resulted in an intermediate phenotype, whereby LC3BII protein levels were lower than those observed with BafA1 alone but higher than those observed with GR-148672X (Fig. 3I).

CytoID cytofluorimetric analysis demonstrated that CES1 blockade by gene knockdown or treatment with GR-148672X also reduced autophagosome vesicle accumulation, confirming the essential role of CES1 in autophagy induction in EOC cells (Fig. 3J-3K). In keeping with the protective role of autophagy during energy stress, treatment with BafA1 exhibited a cytotoxic effect of varying degree in EOC cell lines (Fig. 3L).

Collectively, these data support a model whereby CES1 upregulation by energy stress promotes a metabolic reprogramming of EOC cells toward OXPHOS and autophagy, which enhance EOC cell survival and chemoresistance.

### Oleate supplementation restores LD turnover and EOC cell survival following CES1 inhibition

Lipophagy is a specialized pathway of autophagy that contributes to LD mobilization and plays a major role in lipid turnover during energy stress [37]. The catabolic fate of LDs is size-dependent: small LDs are preferentially degraded via lipophagy, whereas larger ones are processed by lipolytic enzymes [38]. These two pathways are thought to act sequentially, with lipolysis converting larger LDs into smaller ones which can then be targeted by lipophagy [38].

**Fig. 4:**
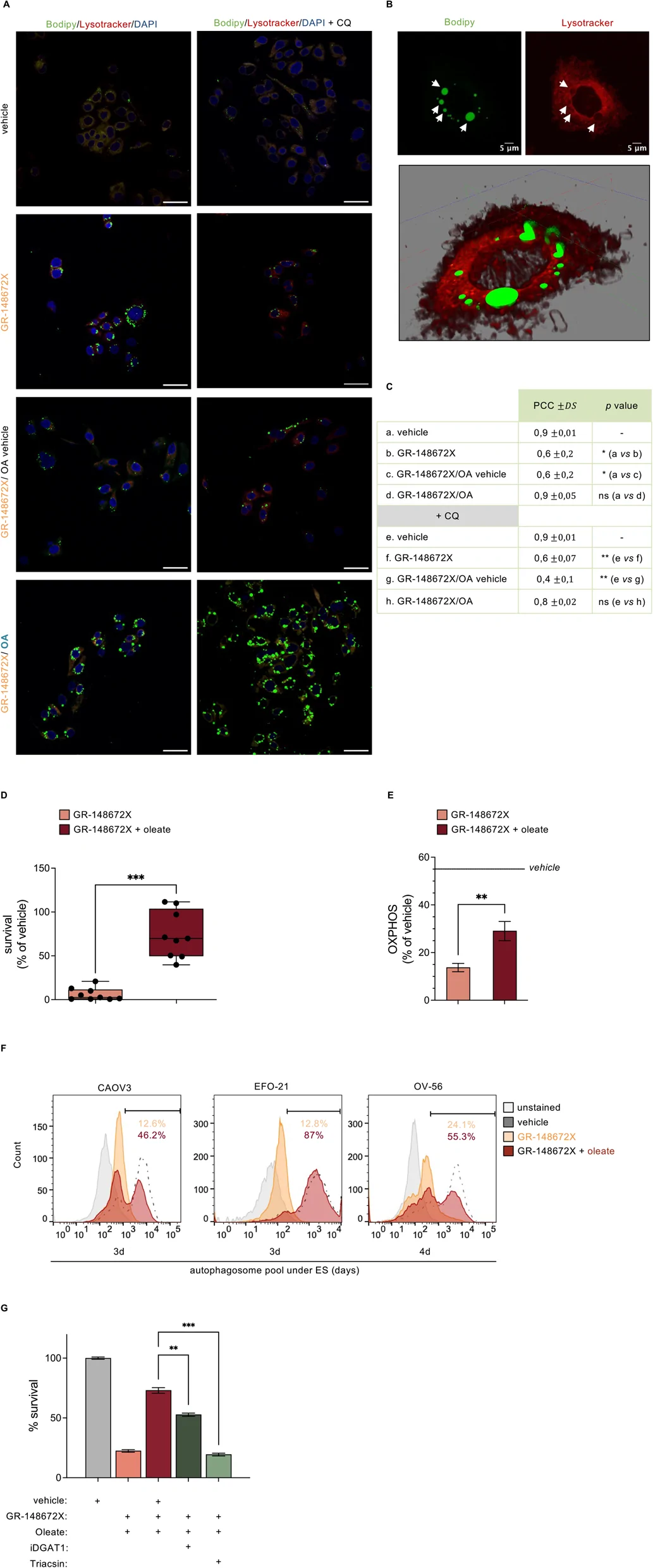
Oleate reverses CES1 blockade–induced defects in lipid droplet dynamics and viability. **A** Representative confocal miscroscopy images of KURAMOCHI cells co-stained with Bodipy 493/503 (neutral lipids in LDs, green), LysoTracker DND 99 (lysosome, red), and DAPI (nuclei, blue) after 2 days of treatment under energy stress in the absence or presence of chloroquine (CQ) for 4 h. Cells were treated with GR-148672X or vehicle alone or in combination with Oleate (OA). **B** Top: representative images of KURAMOCHI treated with GR-148672X in combination with OA, and stained as in A, acquired through the Lightning adaptive de-convolution algorithm with LAS X software. Bigger LDs are indicated by white arrows. Bottom: 3D visualization of acquired series orthogonally sectioned to highlight the lack of colocalization in presence of larger LDs. **C** Quantitative colocalization analysis showing the Pearson''s Correlation Coefficient (PCC) between the green and red channels for each experimental condition as indicated. Data are presented as mean ± SD (*n* = 10 cells per condition). Statistical significance was assessed using a one-way ANOVA test, followed by Holm-Sidak *post-hoc* multiple comparisons test. *, *p* < 0.05, **, *p* < 0.01. **D** CellTiter-Glo® 2.0 viability analysis of EOC cell lines after a 4-day treatment with GR-148672X alone or in combination with oleate under energy stress. **E** Percentage of OXPHOS usage in KURAMOCHI cells treated with GR-148672X alone or in combination with oleate after 3 days of energy stress calculated as in Fig. 1A. **F** Cyto-ID FACS analysis showing autophagosome accumulation in the indicated EOC cells treated with GR-148672X alone or in combination with oleate under energy stress. **G** CellTiter-Glo® 2.0 viability analysis of KURAMOCHI cells assessing the effects of DGAT1 inhibitor or Triacsin after a 4-day treatment under energy stress in combination with GR-148672X and oleate. **D**-**G** Values are shown as mean ± SD. Statistical significance was calculated by 2-tailed Student’s *t* test. **, *p* < 0.01, ***, *p* < 0.001

Since CES1 hydrolyzes neutral lipids stored in LDs [14, 15, 32, 38], we investigated its role in LD dynamics. In the KURAMOCHI cell line, CES1 inhibition significantly impaired lipophagy, leading to an accumulation of larger LDs, as shown by the reduced co-localization of LDs (green) with LysoTracker-stained lysosomes (red). This effect was especially evident upon concomitant lysosomal blockade with chloroquine (CQ) (Fig. 4A-4C, S4A-E). Importantly, oleate supplementation, which replenishes the FFA pool and thereby bypasses the requirement for lipolysis, restored LD degradation by lipophagy (Fig. 4A-C, S4A-E). Notably, oleate supplementation increased the accumulation of LDs of different size. Nevertheless, although the overall co-localization of LDs with lysosomes was comparable to that seen in vehicle-treated cells, larger LDs remained refractory to lipophagy-mediated degradation (Fig. 4B).

Notably, oleate supplementation also restored OXPHOS utilisation, thereby rescuing energy stress-cultured EOC cells from GR-148672X-induced cell death (Fig. 4D-4E). Further, exposure to exogenous oleate reactivated autophagic flux, as shown by the increased autophagosome accumulation observed in GR-148672X-treated cells (Fig. 4F).

Together, these data support a model whereby CES1 sustains lipolysis by breaking down large LDs into smaller ones that can be subsequently degraded by lipophagy. This mechanism alone, however, does not fully explain the ability of exogenous oleate to restore autophagosome accumulation. Thus, we hypothesized that beyond providing FFAs that can be incorporated into TAGs and CEs for storage in smaller LDs, CES1 supports autophagosome biogenesis by supplying FFAs to be esterified into phospholipids for membrane formation in autophagosome assembly. Consistent with this hypothesis, the inhibition of long-chain fatty Acyl-CoA Synthetase (ACSL) isoforms (including ACSL3 and ACSL4) by triacsin C, which prevents the activation of FFAs into acyl-CoAs, a critical step required for phospholipid, TAG and CE synthesis [39], completely abrogated the protective effect of oleate supplementation on cell survival following treatment with GR-148672X (Fig. 4G). In contrast, pharmacologic inhibition of Diacylglycerol O-Acyltransferase 1 (DGAT1), which catalyzes the last enzymatic step in TAG synthesis and is required for LD biogenesis, could only partly rescue EOC cell survival in GR-148672X-treated cells (Fig. 4G). These data suggest that CES1-dependent lipolysis sustains autophagy by providing FFAs not only for TAGs to be stored in LDs, but also for PLs used for membrane biogenesis of autophagosomes.

### CES1 blockade impairs tumor growth in clinically relevant HGSOC models in vivo

**Fig. 5:**
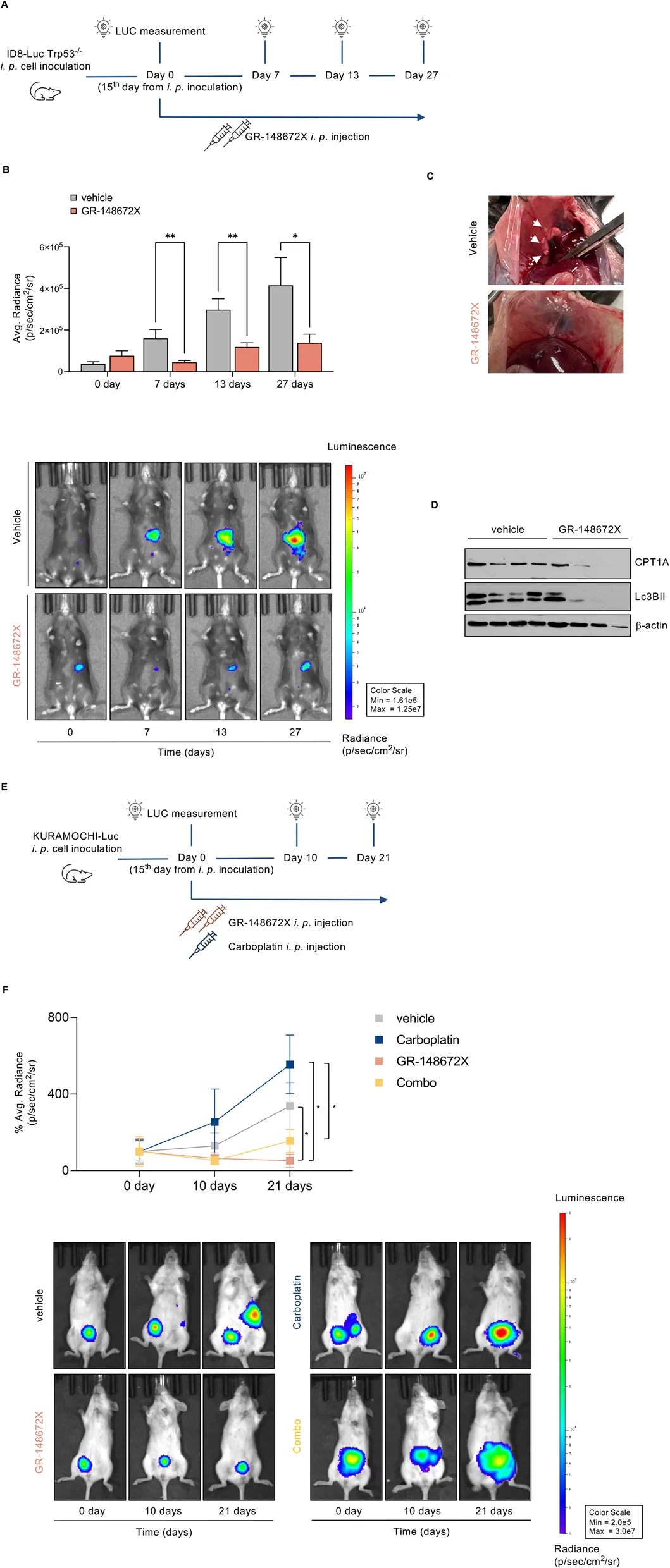
CES1 inhibition reduces HGSOC growth in vivo. **A** Schematic diagram of the in vivo syngeneic mouse model summarizing the schedule of treatment and bioluminescence readings. **B** Bioluminescence measurements expressed as average radiance (p/sec/cm^2^/sr) in ID8-Luc *Trp53*^−/−^ -bearing mice treated with either GR-148672X (50 mg/kg, twice a day) or vehicle via intraperitoneal injection (*i.p.*) (top). Shown are representative images of bioluminescence emission from the same mice at the indicated time points (bottom). Values are shown as mean ± SEM. Statistical significance was calculated by 2-tailed Student’s *t* test. *, *p* < 0.05, **, *p* < 0.01. **C** Representative images of solid tumors on diaphragm (white arrows) found ex vivo in mice from B. **D** Western blots showing CPT1A, LC3BII and β-actin protein levels in mice treated as in B. **E** Schematic diagram of the in vivo xenograft mouse model summarizing the schedule of treatment and bioluminescence readings. **F** Bioluminescence analysis of average radiance (p/sec/cm^2^/sr) in KURAMOCHI-Luc-bearing NSG mice treated with vehicle, GR-148672X (50 mg/kg, *i.p.*, twice a day, every day), Carboplatin (20 mg/kg, *i.p.*, every other day) or their combination (top) at the indicated times. Shown are representative images of bioluminescence emission from each group at the indicated time points (bottom). The results are expressed as percentages with respect to day 0. Statistical significance was calculated by 2-tailed Student’s *t* test. *, *p* < 0.05

To verify the actionability of CES1 as a therapeutic target in EOC, we employed the *Trp53*^−/−^ ID8-Luc orthotopic syngeneic animal model of HGSOC [16]. This model introduces a *Trp53* knockout mutation – a near-universal feature of human HGSOC – by CRISPR/Cas9 gene editing into the ID8-Luc cell line to produce a preclinical experimental system that closely resembles the genetics, biology and clinical evolution of the human disease [16]. As expected, syngeneic *Trp53*^−/−^ ID8-Luc tumors grew rapidly when implanted in the peritoneum of recipient mice (Fig. 5A-B). Strikingly, however, treatment with GR-148672X significantly reduced tumor growth (Fig. 5A-B). Further, whereas 60% of vehicle-treated mice developed macroscopic miliary metastases to the diaphragm, no such metastases were observed in GR-148672X-treated mice (Fig. 5C), indicating that CES1 inhibition effectively impairs both cancer growth and dissemination within the peritoneal cavity, a primary cause of mortality in patients with HGSOC. To investigate the underlying mechanisms, we performed Western blot analyses on protein extracts derived from cells recovered from the peritoneal cavity of mice treated with vehicle or GR-148672X. Notably, GR-148672X treatment was associated with reduced levels of CPT1A and LC3BII, indicating an impairment of oxidative metabolism and autophagy, consistent with the proposed mechanism of CES1-driven metabolic plasticity (Fig. 5D). While future studies specifically designed to monitor survival endpoints will be required to determine whether CES1 inhibition prolongs overall survival in this model, these results extend the findings from previous studies, which showed that genetic or pharmacological CES1 blockade effectively hampers tumor growth in other types of cancer [13, 14, 26]. We next evaluated the therapeutic effect of GR-148672X in a platinum-resistant HGSOC xenograft model generated by intraperitoneal injection of KURAMOCHI-Luc cells into NSG immunocompromised mice. GR-148672X treatment significantly reduced tumor growth compared with both vehicle- and carboplatin-treated animals. Conversely, carboplatin treatment was associated with enhanced tumor progression, consistent with previous observations in the same HGSOC model [24]. Importantly, the combination of GR-148672X and carboplatin significantly reduced tumor burden compared with carboplatin-treated mice, suggesting that CES1 inhibition counteracts tumor-promoting adaptive responses associated with platinum treatment (Fig. 5E–5F). Taken together, these findings further support the therapeutic relevance of CES1 inhibition in HGSOC characterized by poor responsiveness to platinum-based therapy.

### CES1 is a core metabolic vulnerability in HGSOC tumors relying on oxidative metabolism

**Fig. 6:**
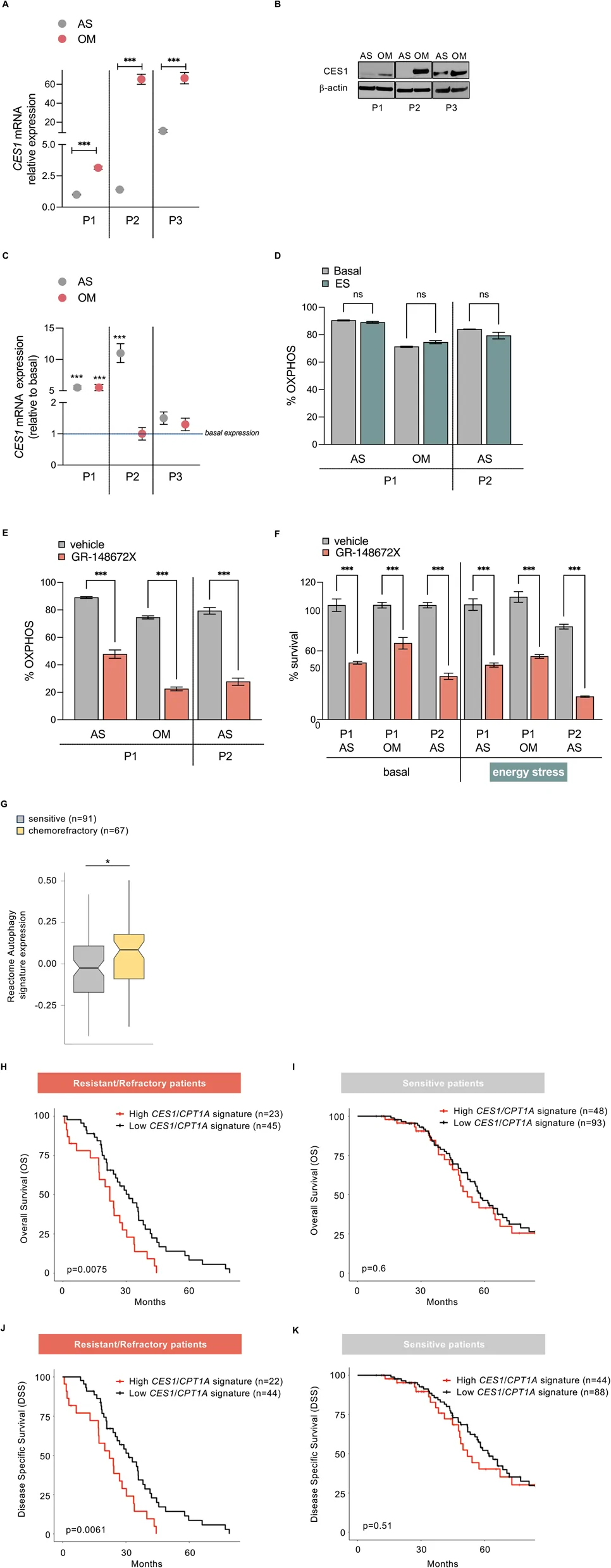
CES1 blockade decreases viability of primary HGSOC cells and high *CES1*/*CPT1A* levels correlate with worse prognosis in refractory patients. **A** qRT-PCR showing *CES1* mRNA levels in three OC patients (P1, P2, P3). Cancer cells were isolated from ascites (AS) and omental metastasis (OM) derived from the same patient and cultured under basal conditions. **B** Western blots showing CES1 protein levels in patients as in A. **C** qRT-PCR showing *CES1* mRNA levels in matched AS- and OM-derived HGSOC cells after 3 days of energy stress compared to basal *CES1* mRNA expression (dotted line). **D**-**E** Percentage of OXPHOS in primary HGSOC cells untreated (D) or treated with GR-148672X or vehicle after 3 days of energy stress (ES) (E) calculated as in Fig. 1A. **F** CellTiter-Glo® 2.0 viability analysis of primary HGSOC cells derived from P1 (AS and OM) and P2 (AS) after 4 days of culture under basal condition or energy stress, and treated with GR-148672X or vehicle. **G** Boxplot showing the mean z-scores of the Reactome Autophagy signature protein expression levels in sensitive (grey, *n* = 91) versus chemorefractory (yellow, *n* = 67) patients. **H**–**K** Overall Survival (OS) curves for chemoresistant/chemorefractory (G, *n* = 68) and sensitive (H, *n* = 141) patients, and Disease-Specific Survival (DSS) curves for chemoresistant/chemorefractory (I, *n* = 66) and sensitive (J, *n* = 132) patients from the UCSC Toil RNA-Seq recompute dataset from The Cancer Genome Atlas (TCGA) Ovarian (OV) program. Patients were stratified based on the average Z-score of the *CES1*/*CPT1A* gene signature, using the first tertile as the threshold. Significance was calculated using the log-rank test. **A,C**-**F** Statistical significance was calculated by 2-tailed Student’s *t* test. Values are shown as mean ± SD (A-C, F) or SEM (D-E). *, *p* < 0.05; ***, *p* < 0.001

To investigate the clinical significance of our findings, we examined the expression of CES1 in matched primary tumor samples derived from ascites (AS) and omental metastases (OM) of patients diagnosed with HGSOC. Interestingly, *CES1* mRNA expression was consistently higher in OM-derived HGSOC cells compared to AS-derived cells (Fig. 6A). Consistent with the mRNA data, CES1 protein levels were increased in OM compared with matched AS samples (Fig. 6B), suggesting a role for CES1 in metastatic spread, in keeping with previous studies demonstrating an involvement of lipid metabolism and FAO in this process [8, 13, 28, 40, 41].

To corroborate our findings in EOC cell lines (see Fig. 2A), we also compared *CES1* expression in primary HGSOC under basal conditions and after energy stress. In samples with relatively low basal *CES1* expression (i.e., AS and OM cells from patient 1 and AS from patient 2), *CES1* was significantly upregulated upon energy stress, whereas its expression remained unchanged in samples exhibiting already high basal *CES1* expression (*i.e.*, OM cells from patient 2 and AS and OM cells from patient 3; Fig. 6C), similar to observations made with EOC cells lines (see Fig. 2A). Further, a metabolic profiling by extracellular flux analysis demonstrated that, irrespective of their anatomical origin, primary HGSOC cells were almost entirely dependent on OXPHOS for ATP production both under basal conditions and during ES (Fig. 6D). Notably, while *CES1* expression levels did not correlate with the percentage of OXPHOS utilization in HGSOC cells, functional CES1 blockade by GR-148672X markedly diminished OXPHOS flux and cell survival across all primary tumor samples (Fig. 6E-6F), indicating an essential role for CES1 in the metabolic wiring of HGSOC cells towards OXPHOS and the ability of these cells to survive in their microenvironment.

In support of the clinical relevance of this CES1-dependent metabolic mechanism in promoting OXPHOS flux and chemoresistance in HGSOC, a re-analysis of the HGSOC patient dataset reported by Chowdhury et al*.* [28] demonstrated a significant upregulation of the autophagy protein signature in chemorefractory compared to chemosensitive tumors (Fig. 6G). The authors themselves previously reported an upregulation of OXPHOS in the same chemorefractory patient cohort [28], thus reinforcing the coordinated contributions of autophagy and oxidative metabolism in promoting platinum chemoresistance in patients.

To further investigate the clinical significance of our findings, we interrogated cohorts of patients with chemosensitive and chemoresistant/chemorefractory tumors from the TCGA HGSOC dataset. For this, we defined a combined *CES1*/*CPT1A* gene signature, since CPT1A has previously been implicated in platinum-resistance in HGSOC [12] and is located downstream of CES1 in the metabolic pathway mobilizing FFAs for FAO. Notably, when patients were stratified based on the tumor-associated expression of this signature, an elevated *CES1*/*CPT1A* gene expression was associated with significantly shorter overall survival (OS) and shorter disease-specific survival (DSS) in the chemoresistant/chemorefractory patient group, but interestingly not in the chemosensitive patient cohort or in the full patient dataset (Fig. 6H-6K, S5A-S5B). Conversely, when analysed independently, neither elevated *CES1* expression, nor *CPT1A* expression alone was associated poorer prognosis in patients with chemoresistant/chemorefractory tumors or in the entire patient cohort (Figure S5C-S5F). Collectively, these data underscore the importance of lipid catabolism and OXPHOS in promoting tumor cell survival under conditions of chemotherapy-exerted pressure and identify CES1 as an actionable therapeutic target to reverse platinum chemoresistance in advanced HGSOC.

## Discussion

Collectively, our findings identify CES1 as a hub in the EOC response to platinum resistance-associated metabolic stress. We find that CES1 is essential for coordinating lipolysis, FAO, and both autophagy and lipophagy to enable EOC cells to cope with metabolic stress and in turn become resistant to platinum-based chemotherapy. These results extend previous studies which demonstrated a role for CES1 in CRC [14] and HCC progression [15], suggesting a broader clinical significance in governing cancer metabolism and serving as a potential therapeutic route for treating chemo-resistant/chemo-refractory HGSOC.

Metabolic reprogramming in the nutrient- deficient TME is a key determinant in cancer cell survival and metastatic dissemination, particularly under selective pressure from anti-cancer therapy, as exemplified by recent studies in platinum-resistant EOC [40].

Accumulating evidence indicates that lipid metabolism plays a central role in chemoresistant settings [12, 26, 39], as the metabolic rewiring toward more energy-demanding pathways seems to be crucial for the expression and function of MDR transporters [12, 42]. Our data indicate that EOC cells adapt to nutrient deprivation by upregulating lipolysis and FAO via CES1 as an alternative source of energy during metabolic stress. *CES1 *expression seems to be regulated at least in part, by NF-κB–dependent transcription, consistent with our previous findings in other tumor contexts [14], although additional stress-responsive or lipid metabolism–related signaling pathways are likely involved*.* In accordance with its role in metabolic adaptation, CES1 inhibition markedly impaired the survival of platinum-resistant cancer cells, reinforcing the idea that targeting lipolysis and FAO could be an effective strategy to overcome resistance in advanced EOC. Notably, FAO has also been linked to aggressive, metastasis-prone disease [8, 41]. HGSOC cells have been shown to rely on FAO to resist anoikis during loss of attachment to the extracellular matrix and spread to ectopic sites in the peritoneal cavity or within the ascitic fluid [8, 41]. FAO also plays an important role in meeting the energy demands of EOC cells in the fat-rich omentum, where tumor cells are in close proximity to and engage in a symbiotic relationship with adipocytes [43]. While there are controversial evidence about the role of oxidative metabolism in chemoresistance [13, 28, 42, 44], our findings are consistent with recent studies which demonstrate that an elevated FAO and OXPHOS are a hallmark of platinum-resistant cell lines, chemoresistant patient-derived xenograft (PDX) tumors, and refractory HGSOCs from patients [13, 28, 42].

Of note, we observed that CES1 inhibition in EOC cells impairs not only OXPHOS, but also autophagic flux. Autophagy is an established survival mechanism in cancer cells, particularly under conditions of metabolic stress [45]. Hence, autophagy-deficient tumors exhibit numerous features that limit their development, including dysfunctional mitochondria, impaired FAO, reduced carbon flux through the TCA cycle, and lipid accumulation that can lead to cytotoxicity [46, 47]. However, the dependence of autophagy on CES1 was surprising. Lipid catabolism is known to regulate multiple cellular processes, beyond energy provision via FAO, including autophagic flux and the formation of autophagosome membranes [48]. Our data support a model whereby CES1-dependent lipolysis promotes autophagy by providing FFAs for formation of complex lipids, including PLs to channel toward membrane biogenesis for the autophagosome assembly via ACSLs enzymes. Hence, CES1 blockade impaired autophagosome biogenesis while exogenous oleate supplementation rescues this phenotype by restoring the cellular FFA pool and cell survival. Further, this protective effect of oleate supplementation was fully reversed by ACSLs inhibition. Notably, ACSL4 has been shown to localise to nascent autophagosomes and regulate autophagy and autophagic flux homeostasis in mammalian cells [49].

We showed that CES1 is also involved in lipophagy. Recent studies have shown that lipolysis is an upstream regulator of lipophagy [48]. In hepatocytes, ATGL-driven lipolysis primarily targets larger LDs, reducing them to smaller LDs that can then be processed by lipophagy [48]. Our findings suggest that CES1-mediated lipolysis similarly influences this process as CES1 inhibition resulted in diminished lipophagy. Oleate supplementation restored lipophagy, likely through its DGAT1/2-mediated esterification into TAGs within smaller LDs. Accordingly, DGAT1 inhibition partially abrogated the protective effect of exogenous oleate on lipophagy suppression by CES1 blockade. Notably, upon CES1 inhibition, EOC cell lines develop a phenotype that resembles that of autophagy-deficient tumors [47, 48], in keeping with an essential role for CES1 in regulating this pathway.

The management of patients with platinum-resistant EOC remains a major clinical challenge, as no effective treatments currently exist for these patients. Except for anti-angiogenic therapy and PARP1 inhibitors, which can only temporarily benefit a select and relatively small subgroup of HGSOC patients, no effective therapies are available for the larger patient population [4, 8]. Recent studies have provided a rationale for targeting autophagy and metabolic reprogramming to overcome cisplatin-resistance in patients with HGSOC [50, 51]. Collectively, our findings suggest that CES1 could provide an actionable therapeutic route for targeting aggressive chemoresistant EOC relying on OXPHOS and autophagy to survive. In support of this idea, we find that CES1 inhibition markedly impairs OXPHOS and cell viability in primary HGSOC cells from patients.

Importantly, GR-148672X also displayed significant antitumor activity in a platinum-resistant xenograft model, where it reduced tumor growth more effectively than carboplatin and significantly attenuated tumor progression associated with platinum treatment. Further, our analyses of human datasets showed a significant upregulation of the autophagy signature in chemorefractory compared to chemosensitive tumors and demonstrated that an elevated *CES1*/*CPT1A* signature portends worse clinical outcomes in patients with chemorefractory HGSOC. Interestingly, we found no association between *CES1*/*CPT1A* signature expression and clinical prognosis in patients with platinum-sensitive tumors, suggesting that increased CES1-dependent lipolysis and FAO may contribute to platinum chemoresistance and hence more aggressive tumor behavior in patients with HGSOC. In line with this hypothesis, the study by Li et al*.* recently reported a strong synergism between cisplatin and CES1 inhibitors in a preclinical model of HCC. They showed how CES1 inhibition resulted in a strong reduction of peroxisome proliferator-activated receptor PPARα/γ/stearoyl-CoA desaturase (SCD) signalling—which is involved in tumor progression and chemoresistance – and that targeting the CES1-PPARα/γ-SCD axis sensitized HCC cells to cisplatin treatment [14]. In contrast, our studies were conducted in a highly platinum-resistant HGSOC setting, in which carboplatin failed to control tumor growth and was associated with enhanced tumor progression, consistent with previous observations in this model [24]. Notably, CES1 inhibition retained robust antitumor activity under these conditions and significantly reduced tumor burden relative to carboplatin-treated animals. Collectively, these findings indicate that CES1 inhibition remains effective despite platinum resistance and highlight CES1 as a promising therapeutic target for disrupting the metabolic plasticity that underpins therapeutic failure in HGSOC.

Over the last few decades, many metabolic mechanisms have been identified as major oncogenic drivers and potential targets for cancer therapy. Previous studies have shown that a number of enzymes involved in lipid metabolism, including CPT1A, fatty acid synthase (FASN), and SCD, are overexpressed in chemoresistant EOC and that pharmacologically blocking these enzymes significantly impairs tumor growth, migration, and tissue invasion both in vitro and in vivo [13, 52]. However, a major barrier to translating these discoveries into patient benefit have been the preclusive on-target toxicities of therapies targeting oxidativemetabolism, given the detrimental effect of these therapies on the metabolic homeostasis in normal tissues. Except for the few-tumours carrying metabolic-gene mutations (*e.g.*, *IDH1/2*), approved-drugs largely target folate-metabolism (methotrexate)/*de-novo* nucleotide-synthesis (5-fluorouracil) [53]. Other early-phase-trials evaluate mitochondrial-complex-I antagonists. However, targeting oncogenic-mitochondrial-metabolism has been fraught with toxicities [53–55]. In contrast, the cytotoxic effects of CES1 inhibition appear to have contextual specificity for cancer cells under conditions of nutrient deprivation, which often present in the TME of advanced and metastatic EOC. Crucially in this respect, pharmacologic CES1 blockade effectively kills cancer cells both in vitro and in vivo without apparent adverse effects. Accordingly, *Ces1d* knockout mice are viable, fertile, and die of old age, indicating that global CES1 deletion is tolerated in vivo [56]. Foremost, the serendipitous identification of healthy individuals carrying homozygous *CES1*-null mutations unequivocally demonstrates the clinical safety of the CES1-targeting approach we propose [57, 58].

## Conclusion

Collectively, our findings demonstrate that CES1 mediates a critical mechanism required for EOC cell survival under conditions of metabolic stress that result in cancer chemo-resistance. We show that CES1 knockdown or pharmacologic inhibition significantly reduces oxidative metabolism, autophagy, and lipophagy, thereby diminishing the viability of platinum-resistant EOC cell lines and primary human HGSOC cells, while impairing EOC growth in vivo, without adverse effects. Importantly, CES1 inhibition retained robust antitumor activity in platinum-resistant HGSOC models, highlighting its ability to target the metabolic adaptations that sustain tumor survival despite platinum-based chemotherapy. These results provide important new insights into the role of CES1-driven metabolic plasticity in enabling EOC cells to withstand chemotherapy. Our data also identify this lipase as an actionable metabolic vulnerability in aggressive platinum-resistant HGSOC, providing a strong rationale for the clinical development of CES1 inhibitors.

## Supplementary Information

Supplementary Material 1.

## Data Availability

All the transcriptomic data used within the paper are publicly available. The UCSC Toil RNA-Seq Recompute data from The Cancer Genome Atlas (TCGA) Ovarian (OV) program were downloaded via the UCSC Xena browser (https://toil.xenahubs.net). Survival and clinical data were downloaded from the Genomic Data Commons repository ( [https://gdc.cancer.gov/node/905/] ). Processed global proteomic data were obtained from the study by Chowdhury et al. [27] and downloaded from the corresponding repository website (https://www.dropbox.com/s/7zul3j1vyrxo40c/processed\_data.zipdl=0).

## Abbreviations

AS: Ascites
CES1: Carboxylesterase 1
CPT1A: Carnitine Palmitoyl-transferase 1A
CQ: Chloroquine
CRC: Colorectal cancer
DGAT: Diacylglycerol O-Acyltransferase 1
DSS: Disease specific survival
EOC: Epithelial ovarian cancer
EOVC: Endometrioid carcinoma
Faa1: Long-chain acyl-coenzyme A synthetase 1
FAO: Fatty acid oxidation
FFA: Free fatty acid
HCC: Hepatocellular carcinoma
HGSOC: High-grade serous carcinoma
LD: Lipid droplets
LGSOC: Low-grade serous carcinoma
MDR: Multidrug resistance
MOC: Mucinous carcinoma
OCCC: Clear-cell carcinoma
OCR: Oxygen consumption rate
OM: Omental metastases
OS: Overall survival
OXPHOS: Oxidative phosphorylation
PARP1: Poly (ADP-ribose) polymerase 1
PDX: Patient-derived xenograft
PPAR: Peroxisome proliferator-activated receptor
TAG: Triacylglycerol
TCGA: The Cancer Genome Atlas
TME: Tumor microenvironment
