# Correction: Carboxylesterase 1 is a core metabolic vulnerability of platinum-resistant high-grade serous ovarian carcinoma

**DOI:** 10.1186/s13046-026-03863-8

**Published:** 2026-11-10

**Authors:** Irene Flati, Davide Vecchiotti, Fiorenza Di Giovanni, Daniela Verzella, Francesca Dall’Aglio, Angelo Sarra, Chiara Battistini, Melissa Zambuto, Giuseppe Frisullo, Jessica Cornice, Ilaria Nunzi, Mauro Di Vito Nolfi, Harry J. Whitwell, Francesca Veglianti, Hafiz Nadeem Khan, Saverio Marchi, Guido Franzoso, Ugo Cavallaro, Daniel D’Andrea, Edoardo Alesse, Francesca Zazzeroni, Daria Capece

**Affiliations:** 1 Department of Biotechnological and Applied Clinical Sciences (DISCAB), University of L’Aquila, L’Aquila, Italy; 2 Microscopy Center, University of L’Aquila, L’ Aquila, Italy; 3 Unit of Gynecological Oncology Research, European Institute of Oncology IRCCS, Milan, Italy; 4 Tumor Immunology and Immunotherapy Unit, IRCCS-Regina ElenaNational Cancer Institute, Rome, Italy; 5 Department of Immunology and Inflammation, Imperial College London, London, UK; 6 Department of Clinical and Molecular Sciences, Polytechnic University, Ancona, Italy; 7 Department of Metabolism, Digestion and Reproduction, Division of Systems Medicine, Imperial College London, London, UK; 8 School of Engineering Mathematics and Technology, University of Bristol, Bristol, UK

Correction: J Exp Clin Cancer Res 45, 217 (2026)

https://doi.org/10.1186/s13046-026-03766-8

Following publication of the original article [1], the authors found that panel labels of Figs. 4, 5 and 6 were mistakenly not processed and not captured in the final version. Specifically, the below panels were missing:

- Figure 4: panels D–G.
- Figure 5: panels E–F.
- Figure 6: panels G–K.

The incorrect and correct figures are presented below. The original article [1] has been corrected.

** Incorrect Fig. **4.

**Fig. 4:**
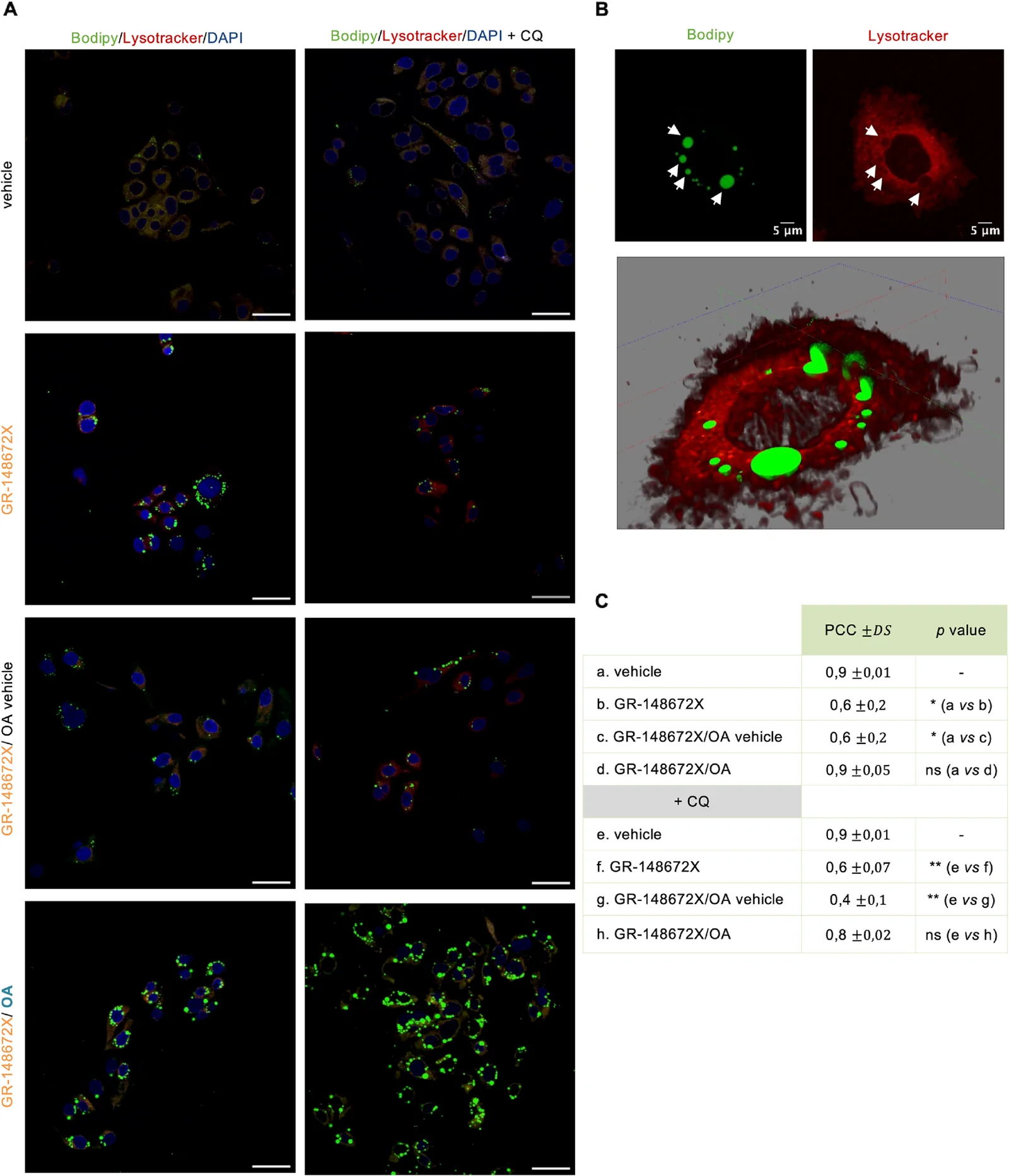
Oleate reverses CES1 blockade–induced defects in lipid droplet dynamics and viability. **A** Representative confocal miscroscopy images of KURAMOCHI cells co-stained with Bodipy 493/503 (neutral lipids in LDs, green), LysoTracker DND 99 (lysosome, red), and DAPI (nuclei, blue) after 2 days of treatment under energy stress in the absence or presence of chloroquine (CQ) for 4 h. Cells were treated with GR-148672X or vehicle alone or in combination with Oleate (OA). **B** Top: representative images of KURAMOCHI treated with GR-148672X in combination with OA, and stained as in A, acquired through the Lightning adaptive de-convolution algorithm with LAS X software. Bigger LDs are indicated by white arrows. Bottom: 3D visualization of acquired series orthogonally sectioned to highlight the lack of colocalization in presence of larger LDs. **C** Quantitative colocalization analysis showing the Pearson’s Correlation Coefficient (PCC) between the green and red channels for each experimental condition as indicated. Data are presented as mean ± SD (*n* = 10 cells per condition). Statistical significance was assessed using a one-way ANOVA test, followed by Holm-Sidak *post-hoc* multiple comparisons test. *, *p* < 0.05, **, *p* < 0.01. **D** CellTiter-Glo^®^ 2.0 viability analysis of EOC cell lines after a 4-day treatment with GR-148672X alone or in combination with oleate under energy stress. **E** Percentage of OXPHOS usage in KURAMOCHI cells treated with GR-148672X alone or in combination with oleate after 3 days of energy stress calculated as in Fig. 1A. **F** Cyto-ID FACS analysis showing autophagosome accumulation in the indicated EOC cells treated with GR-148672X alone or in combination with oleate under energy stress. **G** CellTiter-Glo^®^ 2.0 viability analysis of KURAMOCHI cells assessing the effects of DGAT1 inhibitor or Triacsin after a 4-day treatment under energy stress in combination with GR-148672X and oleate. **D**-**G** Values are shown as mean ± SD. Statistical significance was calculated by 2-tailed Student’s *t* test. **, *p* < 0.01, ***, *p* < 0.001

**Correct Fig. **4.

**Fig. 4:**
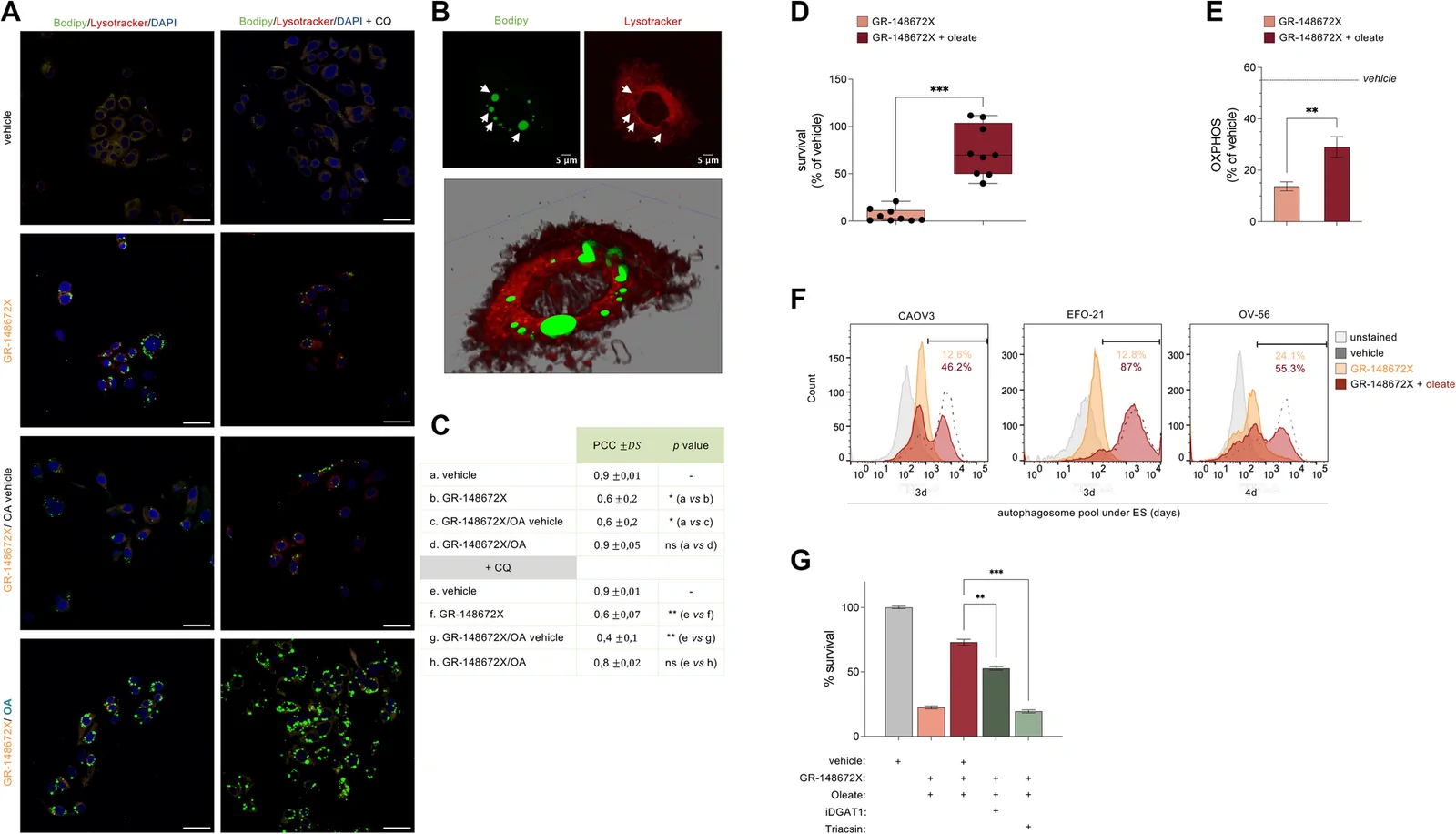
Oleate reverses CES1 blockade–induced defects in lipid droplet dynamics and viability. **A** Representative confocal miscroscopy images of KURAMOCHI cells co-stained with Bodipy 493/503 (neutral lipids in LDs, green), LysoTracker DND 99 (lysosome, red), and DAPI (nuclei, blue) after 2 days of treatment under energy stress in the absence or presence of chloroquine (CQ) for 4 h. Cells were treated with GR-148672X or vehicle alone or in combination with Oleate (OA). **B** Top: representative images of KURAMOCHI treated with GR-148672X in combination with OA, and stained as in A, acquired through the Lightning adaptive de-convolution algorithm with LAS X software. Bigger LDs are indicated by white arrows. Bottom: 3D visualization of acquired series orthogonally sectioned to highlight the lack of colocalization in presence of larger LDs. **C** Quantitative colocalization analysis showing the Pearson’s Correlation Coefficient (PCC) between the green and red channels for each experimental condition as indicated. Data are presented as mean ± SD (n = 10 cells per condition). Statistical significance was assessed using a one-way ANOVA test, followed by Holm-Sidak post-hoc multiple comparisons test. *, *p* < 0.05, **, *p* < 0.01. **D** CellTiter-Glo® 2.0 viability analysis of EOC cell lines after a 4-day treatment with GR-148672X alone or in combination with oleate under energy stress. **E** Percentage of OXPHOS usage in KURAMOCHI cells treated with GR-148672X alone or in combination with oleate after 3 days of energy stress calculated as in Fig. 1A. **F** Cyto-ID FACS analysis showing autophagosome accumulation in the indicated EOC cells treated with GR-148672X alone or in combination with oleate under energy stress. **G** CellTiter-Glo® 2.0 viability analysis of KURAMOCHI cells assessing the effects of DGAT1 inhibitor or Triacsin after a 4-day treatment under energy stress in combination with GR-148672X and oleate. **D**-**G** Values are shown as mean ± SD. Statistical significance was calculated by 2-tailed Student’s t test. **, *p* < 0.01, ***, *p* < 0.001

**Incorrect Fig. **5.

**Fig. 5:**
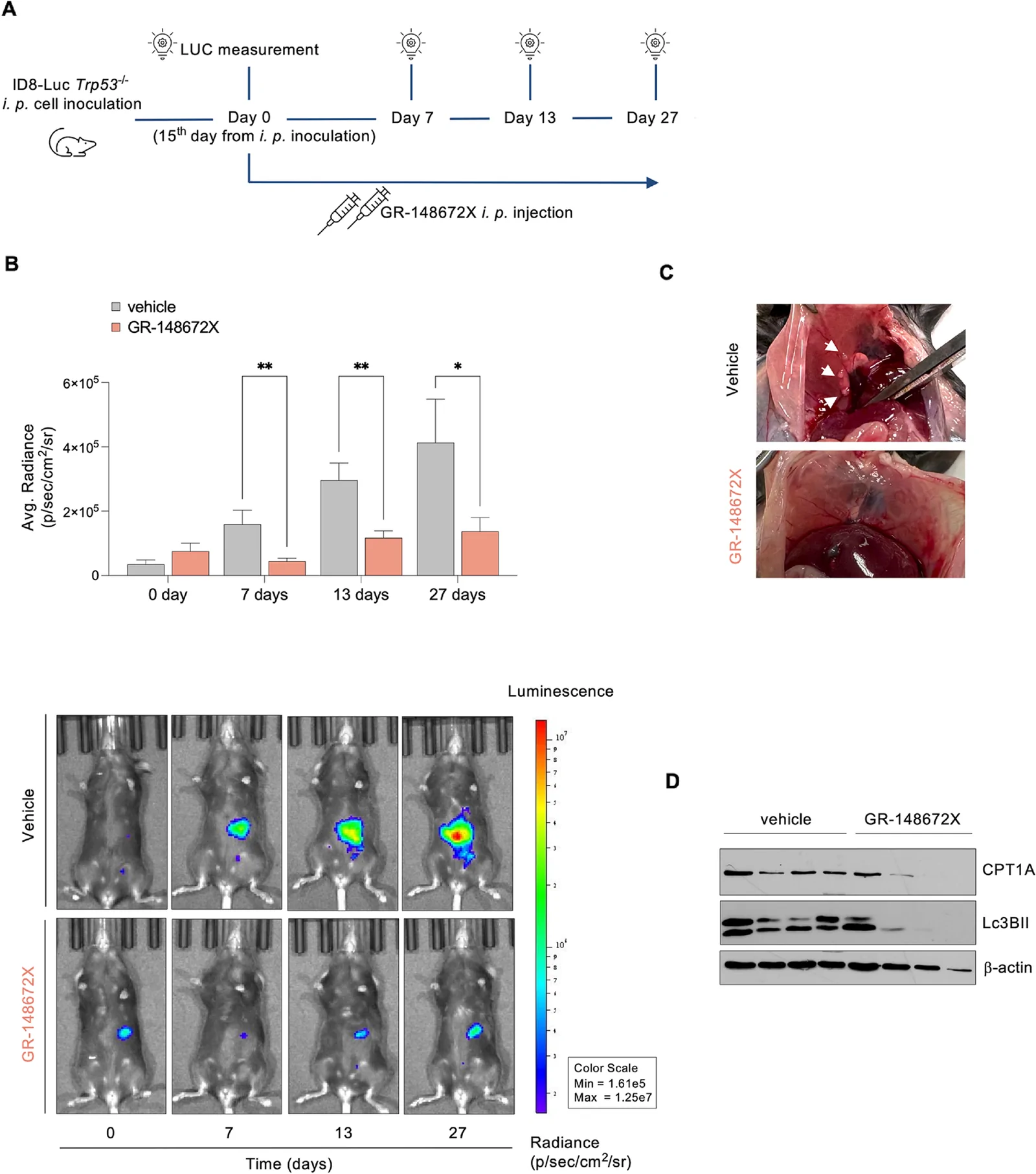
CES1 inhibition reduces HGSOC growth in vivo. **A** Schematic diagram of the in vivo syngeneic mouse model summarizing the schedule of treat-ment and bioluminescence readings. **B** Bioluminescence measurements expressed as average radiance (p/sec/cm^2^/sr) in ID8-Luc *Trp53*^−/−^ -bearing mice treated with either GR-148,672 × (50 mg/kg, twice a day) or vehicle via intraperitoneal injection (*i.p.*) (top). Shown are representative images of biolumi-nescence emission from the same mice at the indicated time points (bottom). Values are shown as mean ± SEM. Statistical significance was calculated by 2-tailed Student’s *t* test. *, *p* < 0.05, **, *p* < 0.01. **C** Representative images of solid tumors on diaphragm (white arrows) found ex vivo in mice from B. **D** West-ern blots showing CPT1A, LC3BII and β-actin protein levels in mice treated as in B. **E**Schematic diagram of the in vivo xenograft mouse model summariz-ing the schedule of treatment and bioluminescence readings. **F** Bioluminescence analysis of average radiance (p/sec/cm^2^/sr) in KURAMOCHI-Luc-bearing NSG mice treated with vehicle, GR-148,672 × (50 mg/kg, *i.p.*, twice a day, every day), Carboplatin (20 mg/kg, *i.p.*, every other day) or their combination (top) at the indicated times. Shown are representative images of bioluminescence emission from each group at the indicated time points (bottom). The results are expressed as percentages with respect to day 0. Statistical significance was calculated by 2-tailed Student’s *t* test. *, *p* < 0.05

**Correct Fig. **5.

**Fig. 5:**
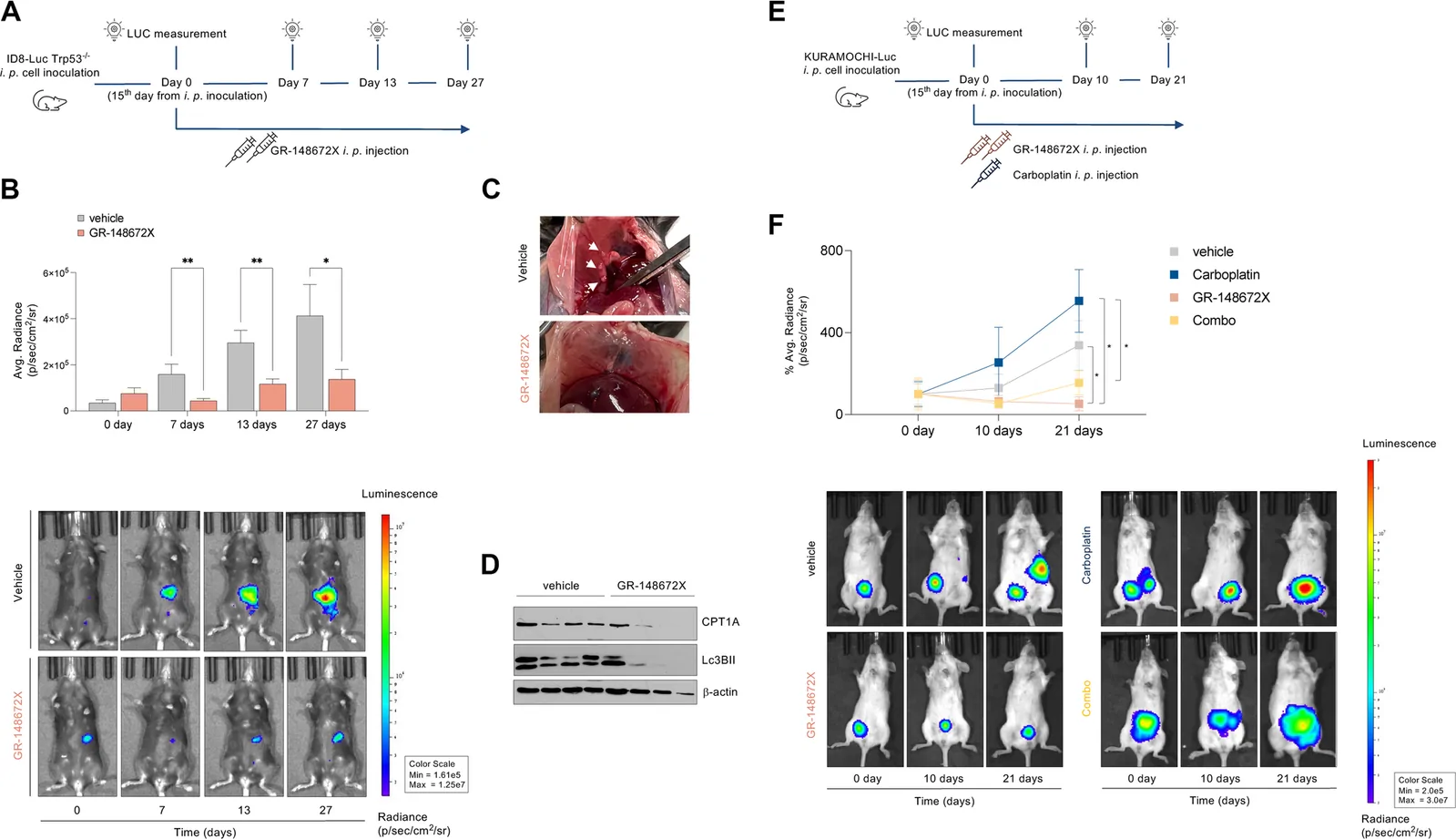
CES1 inhibition reduces HGSOC growth in vivo. **A** Schematic diagram of the in vivo syngeneic mouse model summarizing the schedule of treat-ment and bioluminescence readings. **B** Bioluminescence measurements expressed as average radiance (p/sec/cm2/sr) in ID8-Luc Trp53−/− -bearing mice treated with either GR-148,672 × (50 mg/kg, twice a day) or vehicle via intraperitoneal injection (i.p.) (top). Shown are representative images of biolumi-nescence emission from the same mice at the indicated time points (bottom). Values are shown as mean ± SEM. Statistical significance was calculated by 2-tailed Student’s t test. *, *p* < 0.05, **, *p* < 0.01. **C** Representative images of solid tumors on diaphragm (white arrows) found ex vivo in mice from B. D West-ern blots showing CPT1A, LC3BII and β-actin protein levels in mice treated as in B. **E **Schematic diagram of the in vivo xenograft mouse model summariz-ing the schedule of treatment and bioluminescence readings. **F** Bioluminescence analysis of average radiance (p/sec/cm2/sr) in KURAMOCHI-Luc-bearing NSG mice treated with vehicle, GR-148,672 × (50 mg/kg, i.p., twice a day, every day), Carboplatin (20 mg/kg, i.p., every other day) or their combination (top) at the indicated times. Shown are representative images of bioluminescence emission from each group at the indicated time points (bottom). The results are expressed as percentages with respect to day 0. Statistical significance was calculated by 2-tailed Student’s t test. *, *p* < 0.05

**Incorrect Fig. **6.

**Fig. 6:**
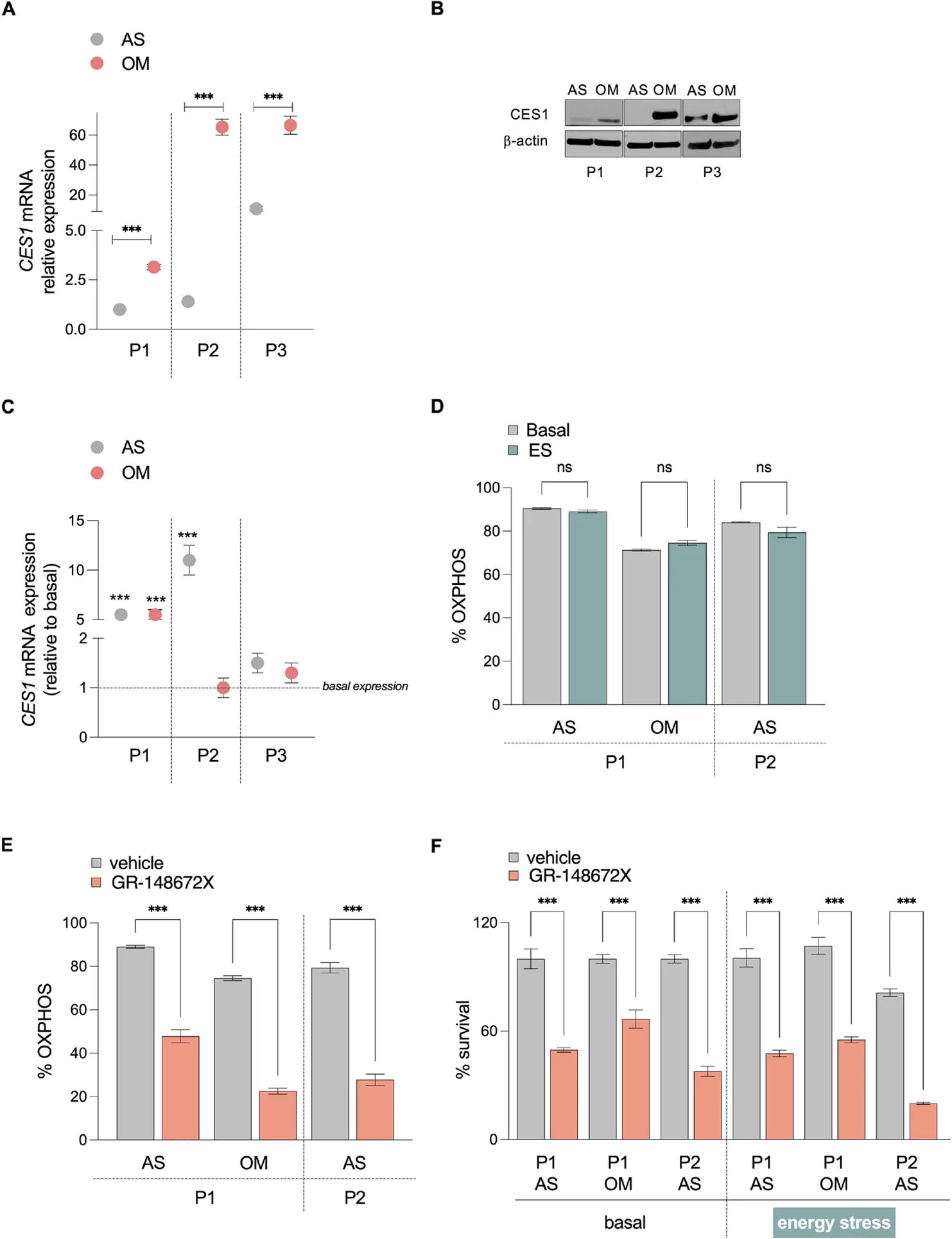
CES1 blockade decreases viability of primary HGSOC cells and high *CES1*/*CPT1A* levels correlate with worse prognosis in refractory patients. **A** qRT-PCR showing *CES1* mRNA levels in three OC patients (P1, P2, P3). Cancer cells were isolated from ascites (AS) and omental metastasis (OM) derived from the same patient and cultured under basal conditions. **B** Western blots showing CES1 protein levels in patients as in A. **C** qRT-PCR showing *CES1* mRNA levels in matched AS- and OM-derived HGSOC cells after 3 days of energy stress compared to basal *CES1* mRNA expression (dotted line). **D**-**E** Percentage of OXPHOS in primary HGSOC cells untreated (D) or treated with GR-148672X or vehicle after 3 days of energy stress (ES) (E) calculated as in Fig. 1A. **F** CellTiter-Glo^®^ 2.0 viability analysis of primary HGSOC cells derived from P1 (AS and OM) and P2 (AS) after 4 days of culture under basal condition or energy stress, and treated with GR-148672X or vehicle. **G** Boxplot showing the mean z-scores of the Reactome Autophagy signature protein expression levels in sensitive (grey, *n* = 91) versus chemorefractory (yellow, *n* = 67) patients. **H**–**K** Overall Survival (OS) curves for chemoresistant/chemorefractory (G, *n* = 68) and sensitive (H, *n* = 141) patients, and Disease-Specific Survival (DSS) curves for chemoresistant/chemorefractory (I, *n* = 66) and sensitive (J, *n* = 132) patients from the UCSC Toil RNA-Seq recompute dataset from The Cancer Genome Atlas (TCGA) Ovarian (OV) program. Patients were stratified based on the average Z-score of the *CES1*/*CPT1A* gene signature, using the first tertile as the threshold. Significance was calculated using the log-rank test. **A**,** C**-**F** Statistical significance was calculated by 2-tailed Student’s *t* test. Values are shown as mean ± SD (A-C, F) or SEM (D-E). *, *p* < 0.05; ***, *p* < 0.0

**Correct Fig. **6.

**Fig. 6:**
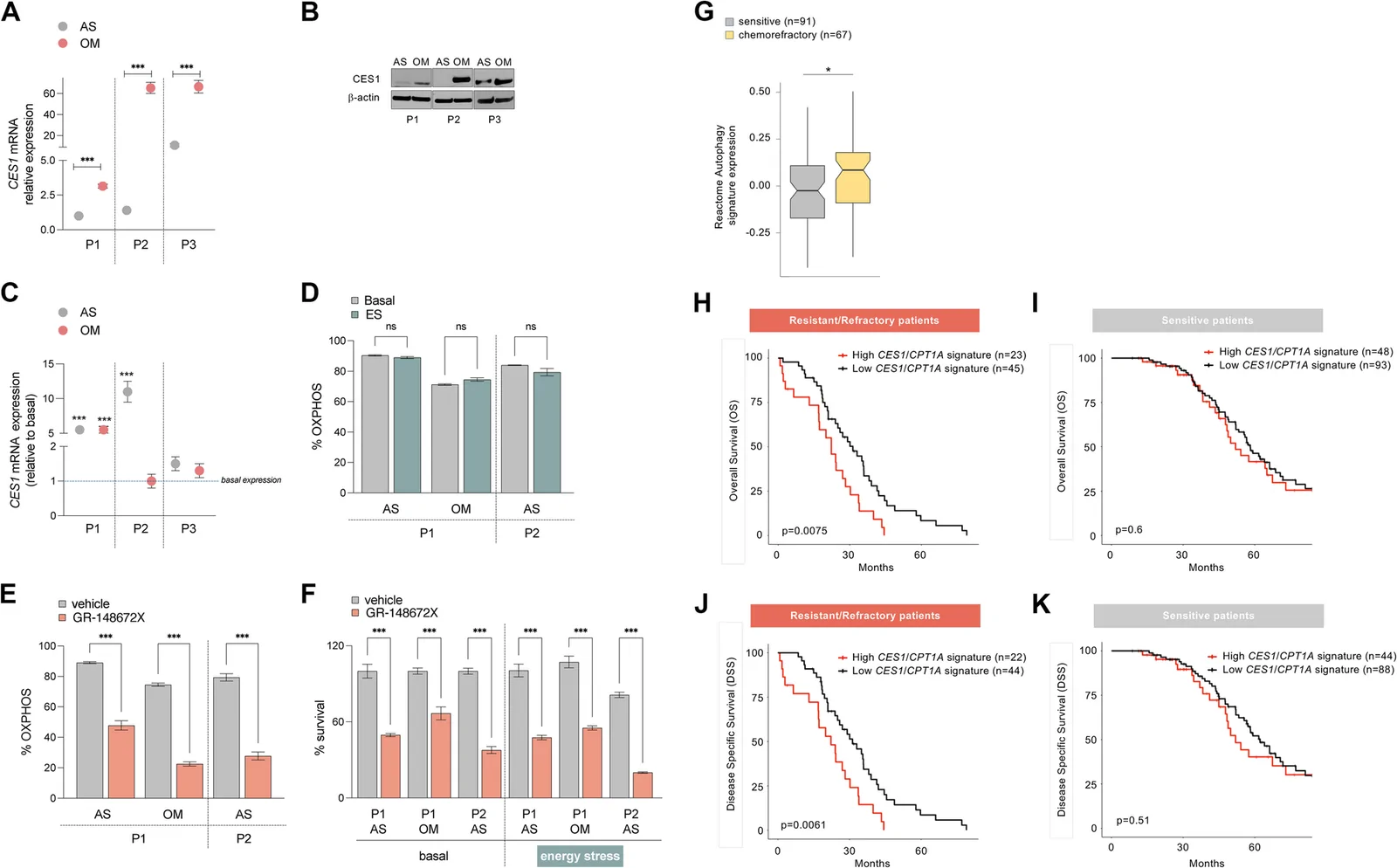
CES1 blockade decreases viability of primary HGSOC cells and high CES1/CPT1A levels correlate with worse prognosis in refractory patients. **A** qRT-PCR showing CES1 mRNA levels in three OC patients (P1, P2, P3). Cancer cells were isolated from ascites (AS) and omental metastasis (OM) derived from the same patient and cultured under basal conditions. **B** Western blots showing CES1 protein levels in patients as in A. **C** qRT-PCR showing CES1 mRNA levels in matched AS- and OM-derived HGSOC cells after 3 days of energy stress compared to basal CES1 mRNA expression (dotted line). **D**-**E** Percentage of OXPHOS in primary HGSOC cells untreated (D) or treated with GR-148672X or vehicle after 3 days of energy stress (ES) (E) calculated as in Fig. 1A. **F** CellTiter-Glo® 2.0 viability analysis of primary HGSOC cells derived from P1 (AS and OM) and P2 (AS) after 4 days of culture under basal condition or energy stress, and treated with GR-148672X or vehicle. **G** Boxplot showing the mean z-scores of the Reactome Autophagy signature protein expression levels in sensitive (grey, n = 91) versus chemorefractory (yellow, n = 67) patients. **H**–**K** Overall Survival (OS) curves for chemoresistant/chemorefractory (G, n = 68) and sensitive (H, n = 141) patients, and Disease-Specific Survival (DSS) curves for chemoresistant/chemorefractory (I, n = 66) and sensitive (J, n = 132) patients from the UCSC Toil RNA-Seq recompute dataset from The Cancer Genome Atlas (TCGA) Ovarian (OV) program. Patients were stratified based on the average Z-score of the CES1/CPT1A gene signature, using the first tertile as the threshold. Significance was calculated using the log-rank test. **A**, **C**-**F** Statistical significance was calculated by 2-tailed Student’s t test. Values are shown as mean ± SD (**A**-**C**, **F**) or SEM (**D**-**E**). *, *p* < 0.05; ***, *p* < 0.0

## References

[1] Flati I, Vecchiotti D, Di Giovanni F, et al. Carboxylesterase 1 is a core metabolic vulnerability of platinum-resistant high-grade serous ovarian carcinoma. J Exp Clin Cancer Res. 2026;45:217. 10.1186/s13046-026-03766-8 PMC13660344 42870294

