# Supplementary material for: Carboxylesterase 1 is a core metabolic vulnerability of platinum-resistant high-grade serous ovarian carcinoma
Source: J Exp Clin Cancer Res. 2026 Jul 24;45:217. doi: 10.1186/s13046-026-03766-8 (PMC13660344; doi:10.1186/s13046-026-03766-8)
Supplement: Supplementary file 1 [file 13046_2026_3766_MOESM1_ESM.pdf]

SUPPLEMENTARY INFORMATION

SUPPLEMENTARY FIGURES AND FIGURE LEGENDS

SUPPLEMENTARY FIGURE 1

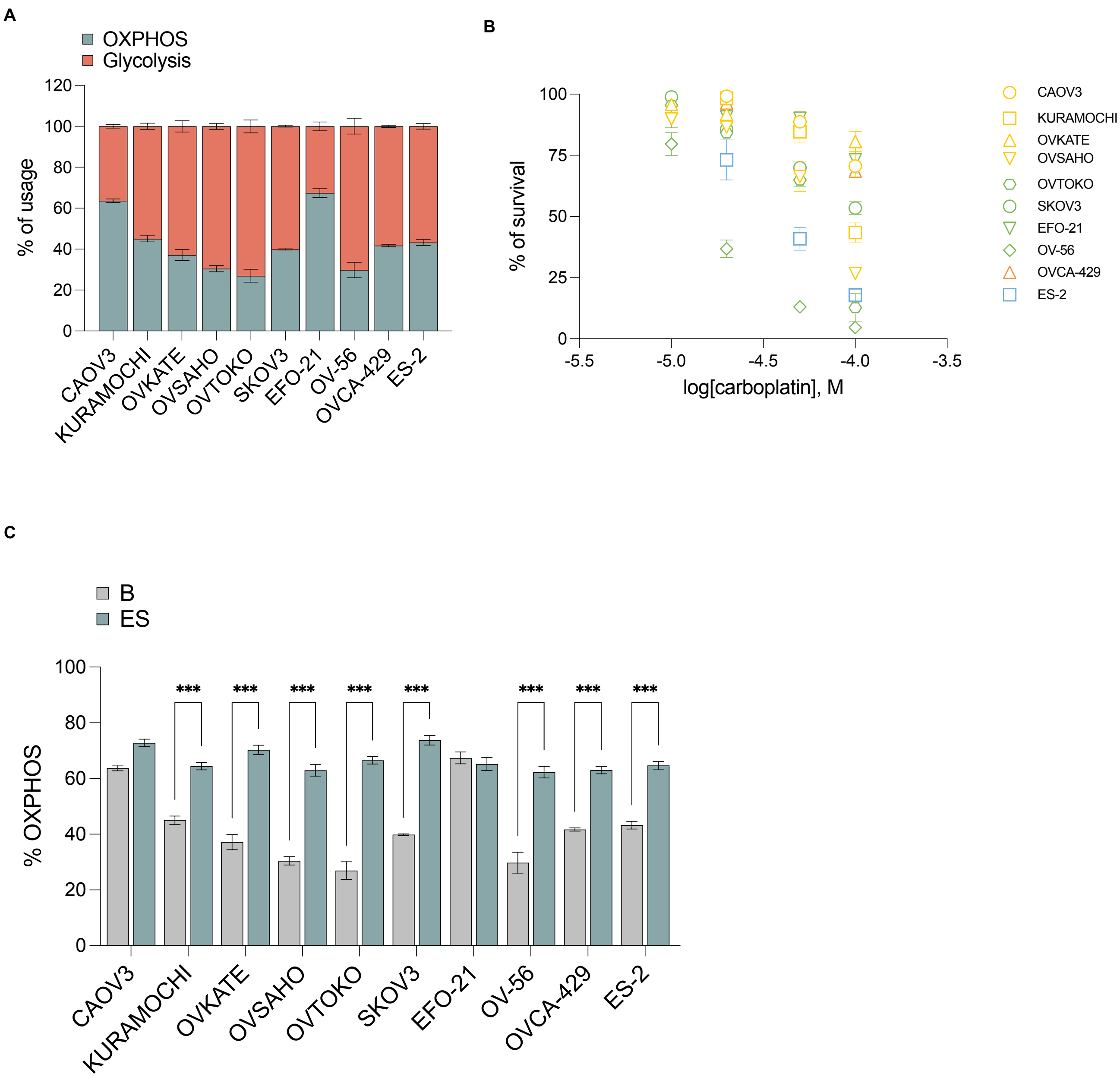

Figure S1 - Related to Figure 1

A] Percentage of OXPHOS and glycolysis usage measured by Seahorse XFe96 ATP rate test in the indicated EOC cells cultured in basal conditions. **B]** Percentage of survival in the indicated EOC cells after 3-day treatment with increasing doses of carboplatin. **C]** Percentage of OXPHOS used by EOC cells under basal (B) and energy stress (ES) condition measured as in A. **A, C]** Values are shown as mean ± SEM. **B]** Values are shown as mean ± SD. **C]** Statistical significance was calculated by 2-tailed Student's *t* test. ***, $p < 0.001$.

SUPPLEMENTARY FIGURE 2

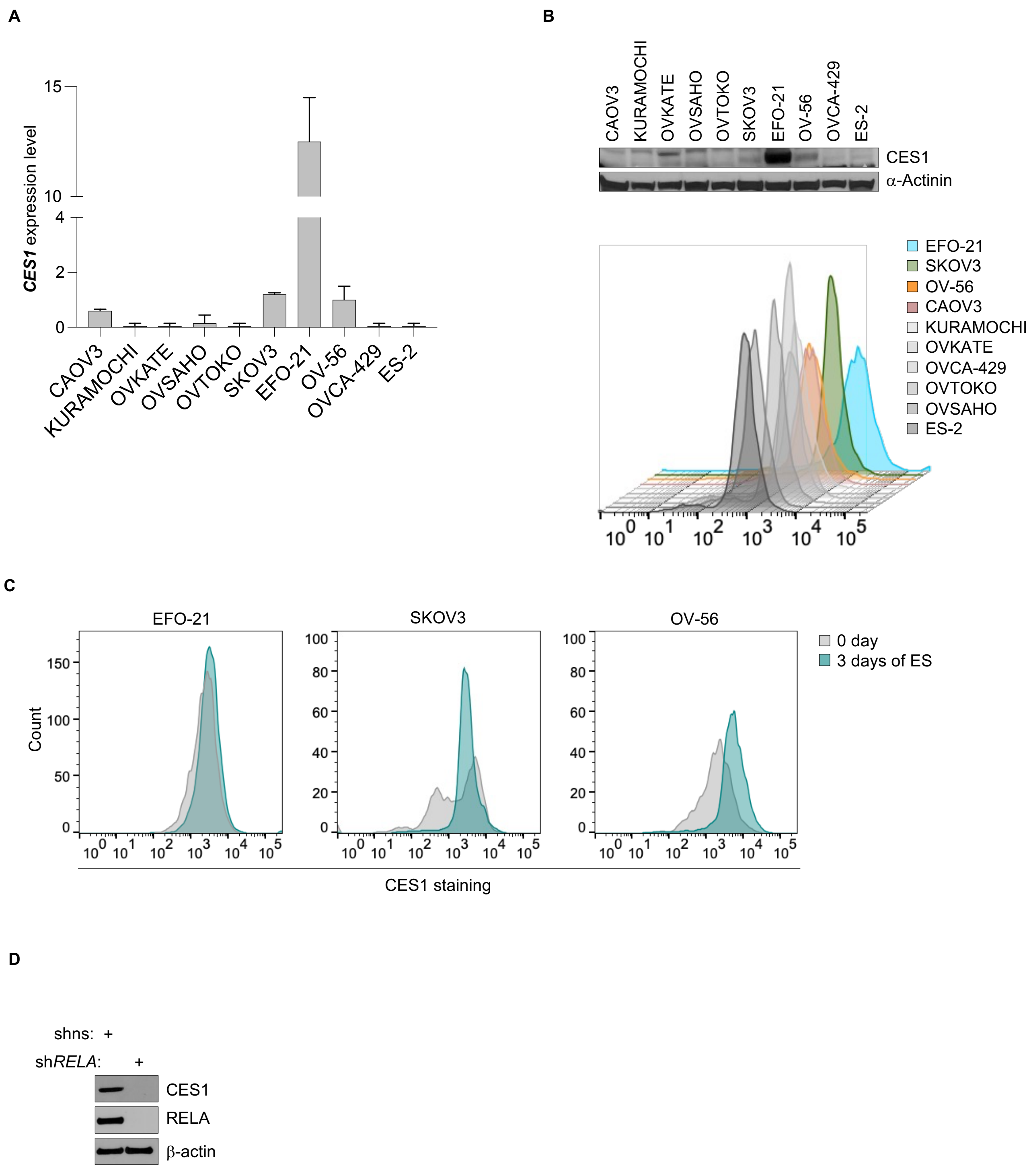

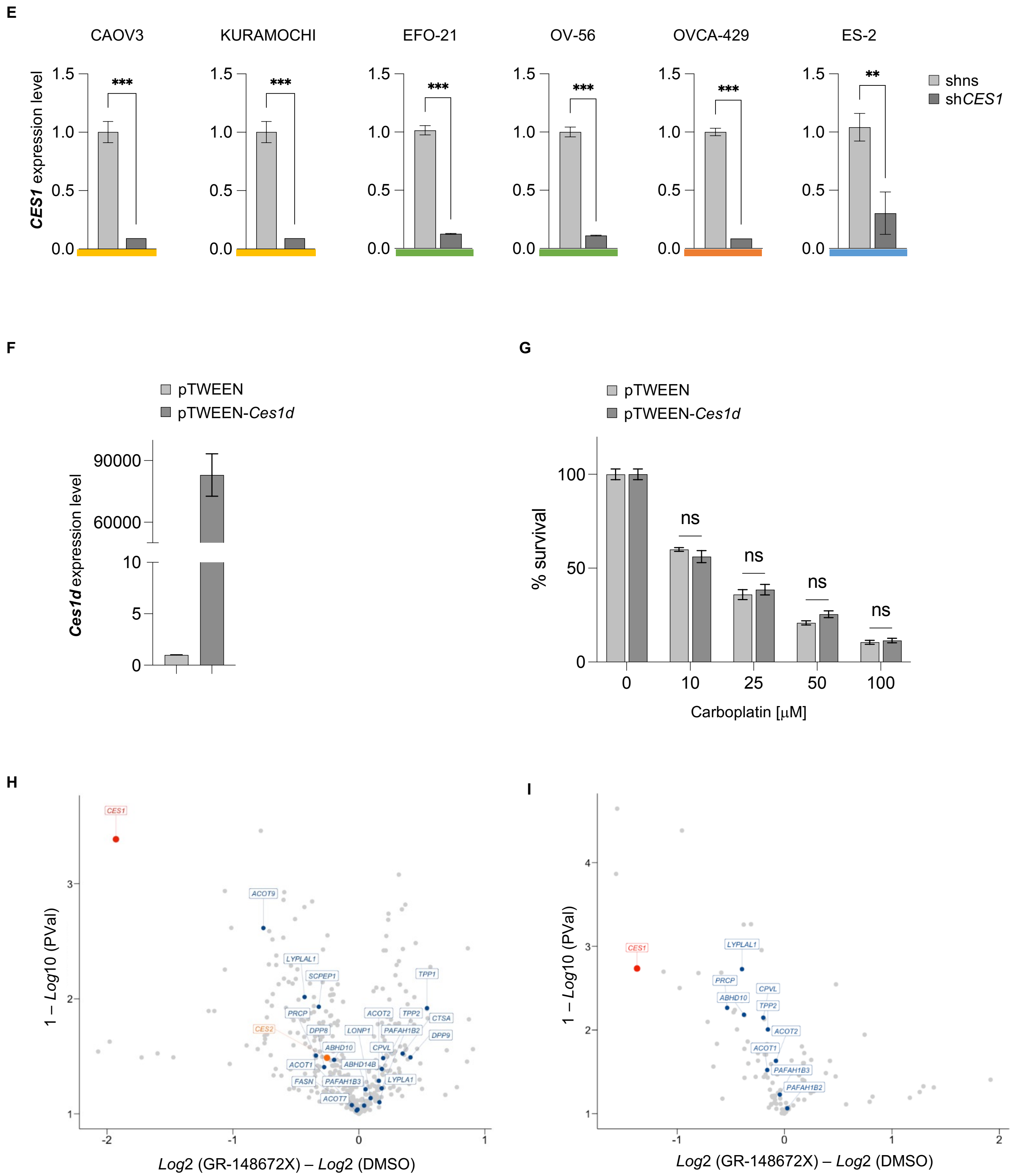
E
CAOV3
KURAMOCHI
EFO-21
OV-56
OVCA-429
ES-2
CES1 expression level
1.5
1.0
0.5
0.0
***
**
shns
shCES1
F
pTWEEN
pTWEEN-Ces1d
Ces1d expression level
90000
60000
10
5
0
G
pTWEEN
pTWEEN-Ces1d
% survival
100
50
0
ns
0
10
25
50
100
Carboplatin [μM]
H
1 – Log10 (PVal)
Log2 (GR-148672X) – Log2 (DMSO)
CES1
CES2
ACOT9
LYPLAL1
SCPEP1
TPP1
PRCP
ACOT2
TPP2
CTSA
DPP8
LONP1
PAFAH1B2
DPP9
ABHD10
CPVL
ACOT1
ABHD14B
FASN
PAFAH1B3
LYPLA1
ACOT7
I
1 – Log10 (PVal)
Log2 (GR-148672X) – Log2 (DMSO)
CES1
LYPLAL1
PRCP
CPVL
ABHD10
TPP2
ACOT2
ACOT1
PAFAH1B3
PAFAH1B2

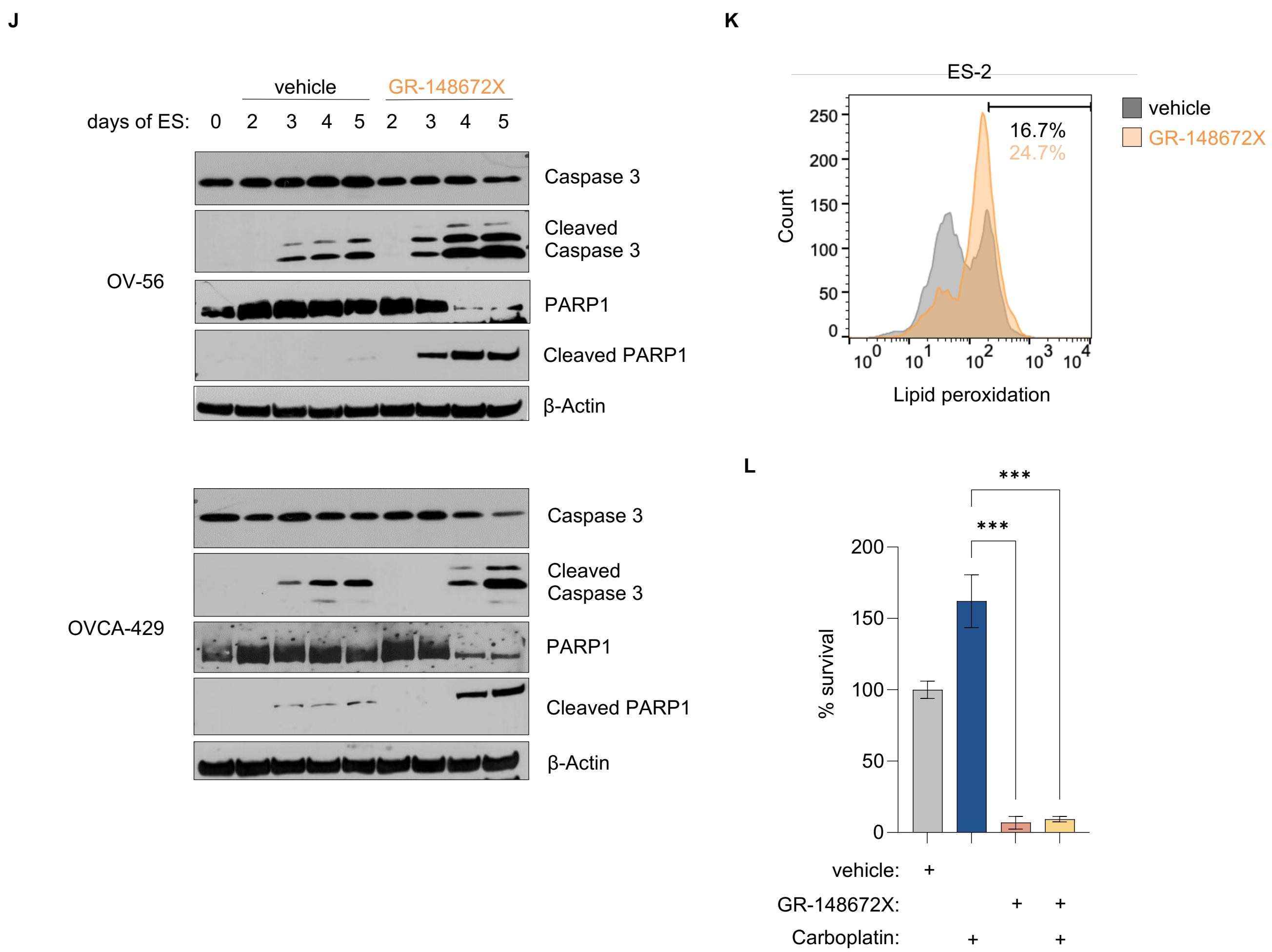

Figure S2 - Related to Figure 2

A] qRT-PCR showing basal *CES1* mRNA levels in the selected panel of ten EOC cells. **B]** CES1 protein levels analyzed by western blot (top) and FACS (bottom) across the selected panel of EOC cell lines. **C]** FACS analyses showing CES1 intracellular staining in the indicated EOC cells at day 0 under basal condition and after 3 days of energy stress (ES). **D]** Western blots showing CES1, RELA and β-actin protein levels in OV-56 cells expressing RELA-specific versus ns shRNA and cultured under normal conditions. **E]** qRT-PCR showing *CES1* mRNA levels in EOC cells expressing sh*CES1* or shns. **F]** qRT-PCR showing *Ces1d* mRNA levels in ID8-Luc Trp53$^{-/-}$ expressing pTWEEN or pTWEEN-*Ces1d*. **G]** CellTiter-Glo® 2.0 viability

analysis of ID8 Luc Tpr53$^{-/-}$ expressing pTWEEN or pTWEEN-*Ces1d* after 4 days of culture under basal condition in presence of increasing doses of Carboplatin (0, 10, 25, 50, 100 mM). **H-I]** On-target CES1-selective specificity of GR-148672X against serine hydrolases expressed in HepG2 and EFO-21 cells, respectively. Differential enrichment of human serine hydrolases following activity-based protein profiling (ABPP) with ActivX® Desthiobiotin-FP probes is shown. The x-axis represents the $\log_2$ fold change (GR-148672X vs. DMSO), and the y-axis shows the $-\log_{10}$(p-value). Blue dots are the annotated serine hydrolases. CES1 and CES2 are indicated in red and orange respectively. **J]** Western blots showing the protein levels of caspase 3, cleaved caspase 3, PARP1, and cleaved PARP1 in the indicated cells treated with GR-148672X. b-actin is shown as loading control. **K]** Lipid peroxidation in ES-2 cells after 4 days of GR-148672X treatment under energy stress. **L]** CellTiter-Glo® 2.0 viability analysis of KURAMOCHI cells treated with GR-148672X in combination with Carboplatin, where indicated, after 3 days of culture under energy stress. **E, F, L]** Values are shown as mean ± SD. Statistical significance was calculated by 2-tailed Student's *t* test. **, $p < 0.01$; ***, $p < 0.001$.

SUPPLEMENTARY FIGURE 3

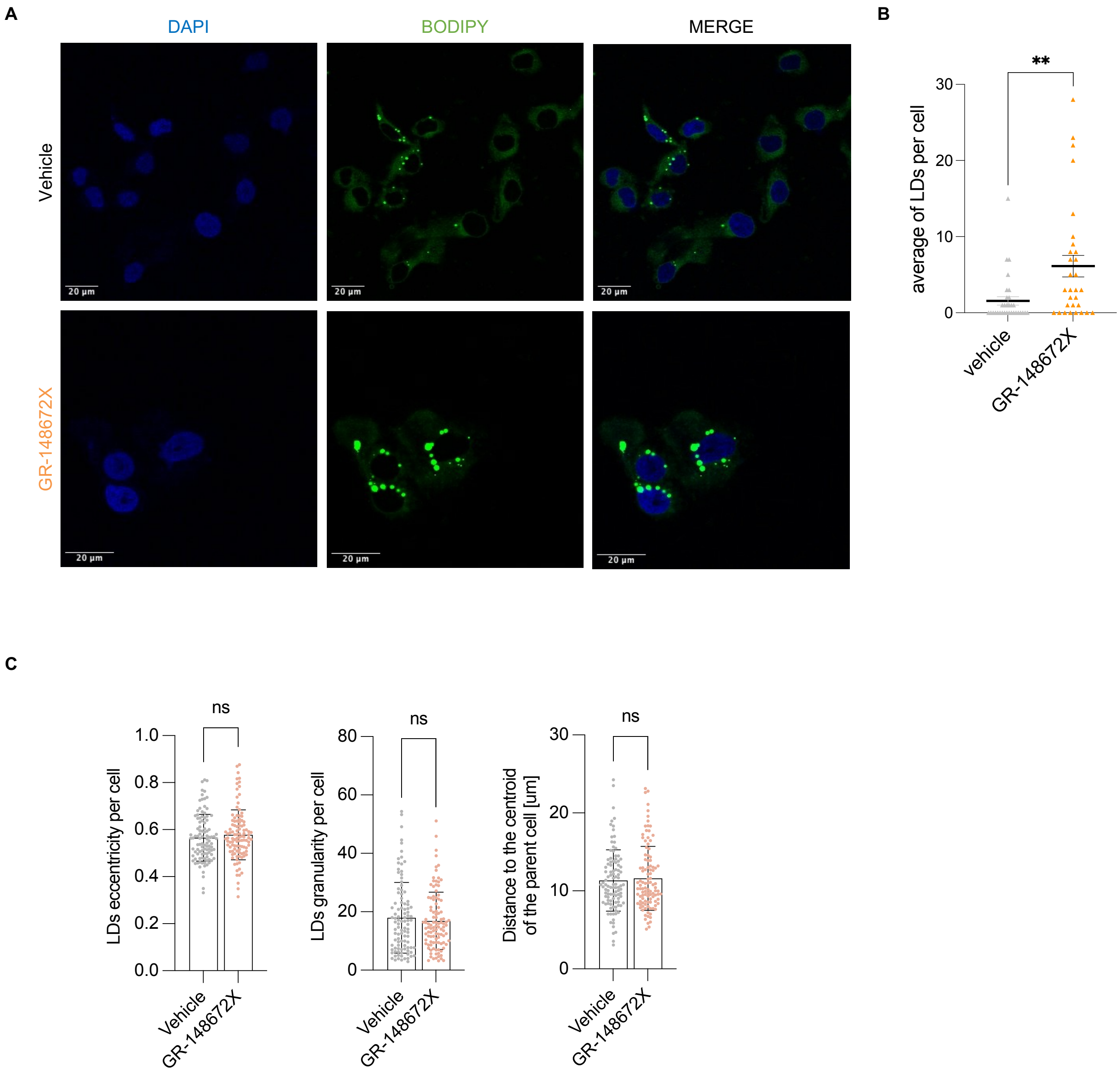

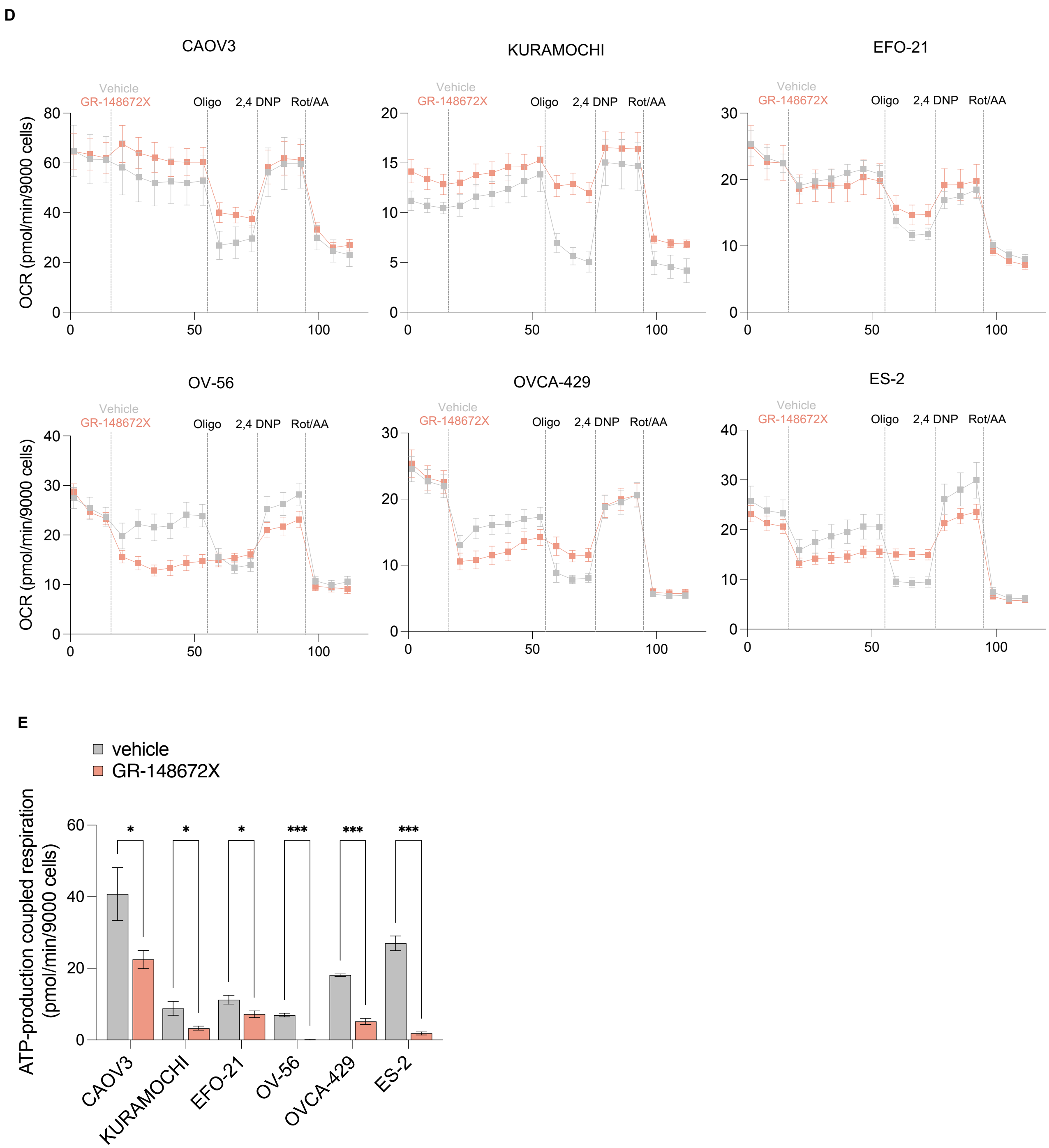

Figure S3 - Related to Figure 3

A] Representative confocal microscopy images of KURAMOCHI cells stained with Bodipy 493/503 (green), and DAPI (nuclei, blue) after 2 days of treatment with GR-148672X or vehicle under energy stress. **B]** Average of LDs per cell count quantified using ImageJ from

KURAMOCHI cells treated as in (A). **C]** Quantitative analysis of LD metrics by Smart Lipid Droplet Digital Assay showing in cells treated as in A **D]** Oxygen consumption rate (OCR) profiling in EOC cells treated with GR-148672X or vehicle after 3 days of energy stress. **E]** Shown is the ATP production-coupled respiration in the indicated EOC cells treated as in B. Values are shown as mean ± SEM. *, $p<0.05$; ***, $p< 0.001$.

SUPPLEMENTARY FIGURE 4

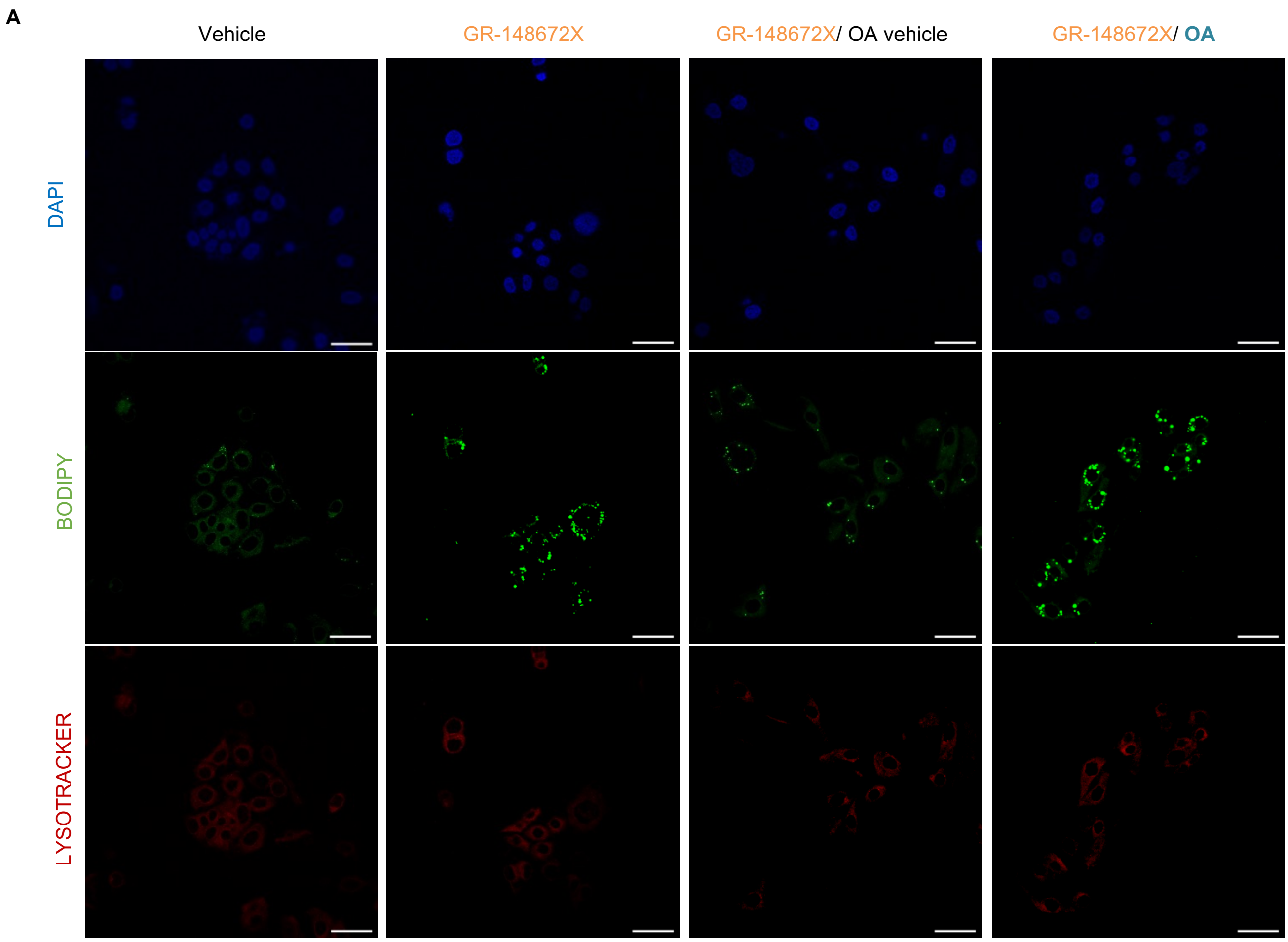

B

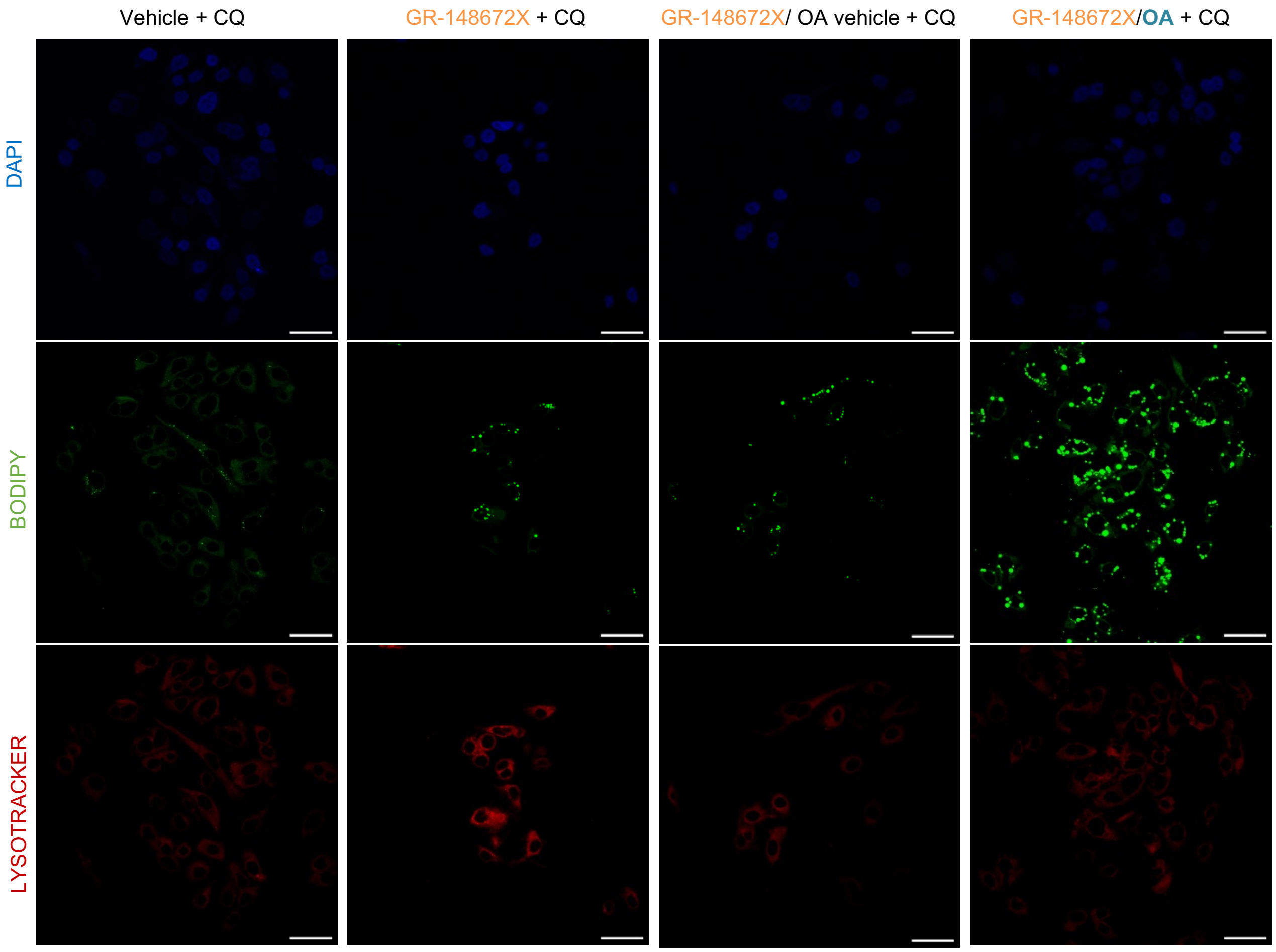
Vehicle + CQ
GR-148672X + CQ
GR-148672X/ OA vehicle + CQ
GR-148672X/OA + CQ
DAPI
BODIPY
LYSOTRACKER

C

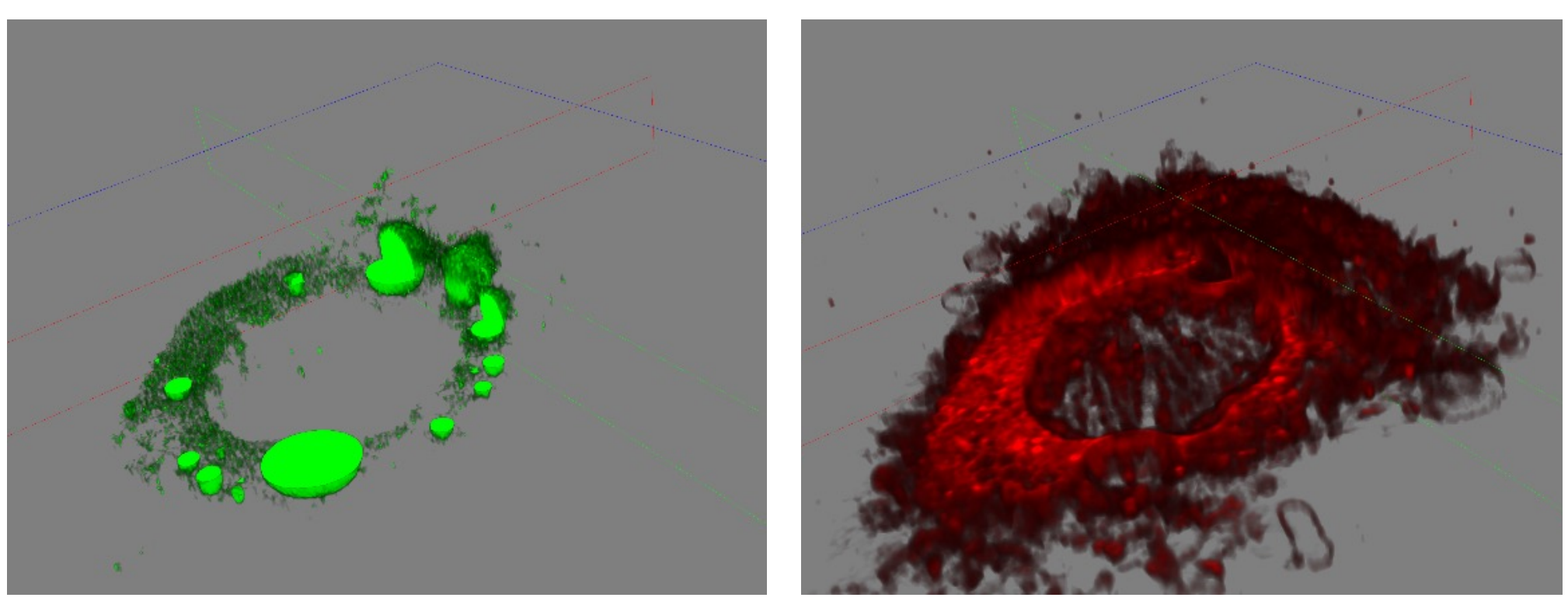

D

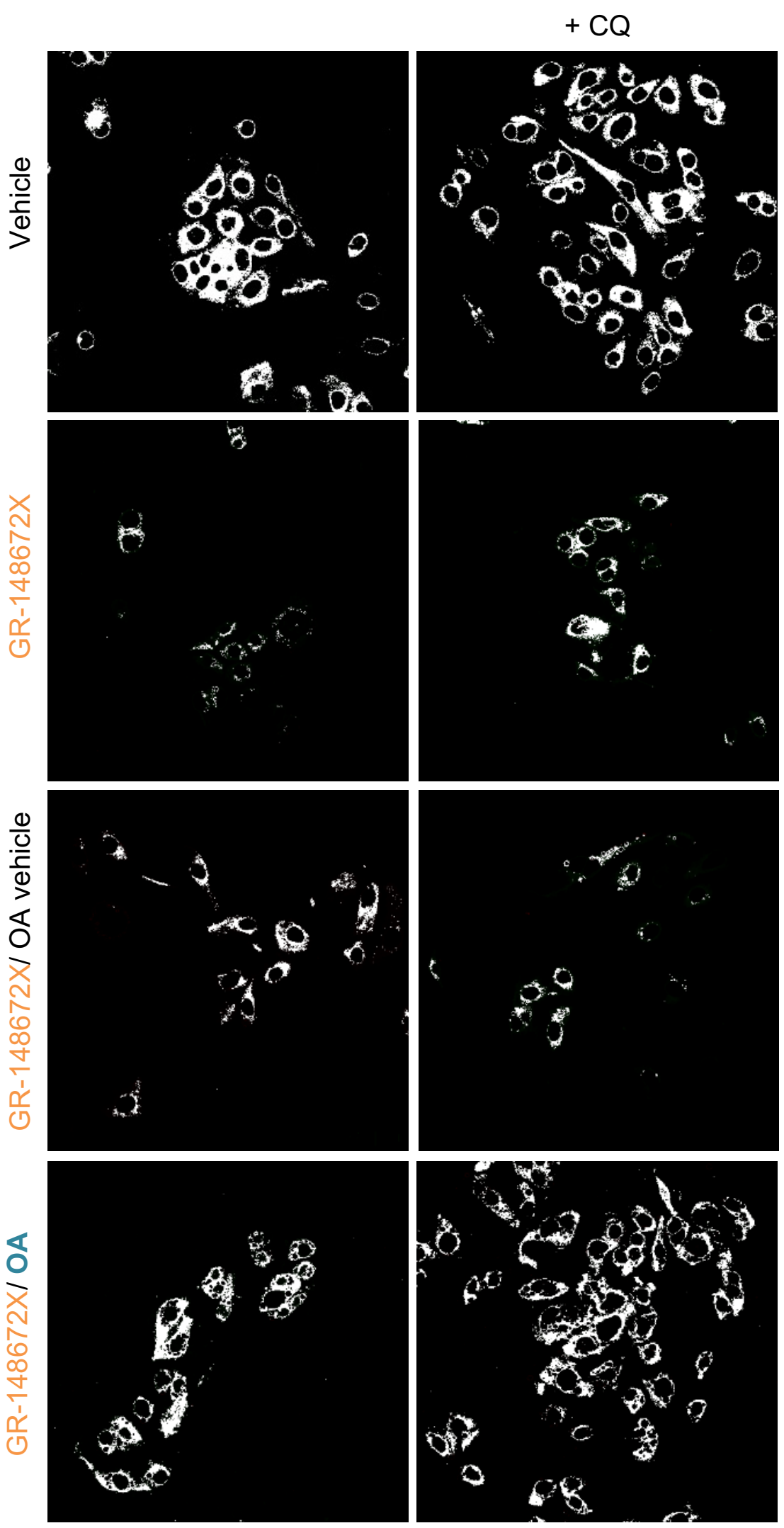

+ CQ
Vehicle
GR-148672X
GR-148672X/ OA vehicle
GR-148672X/ OA

E

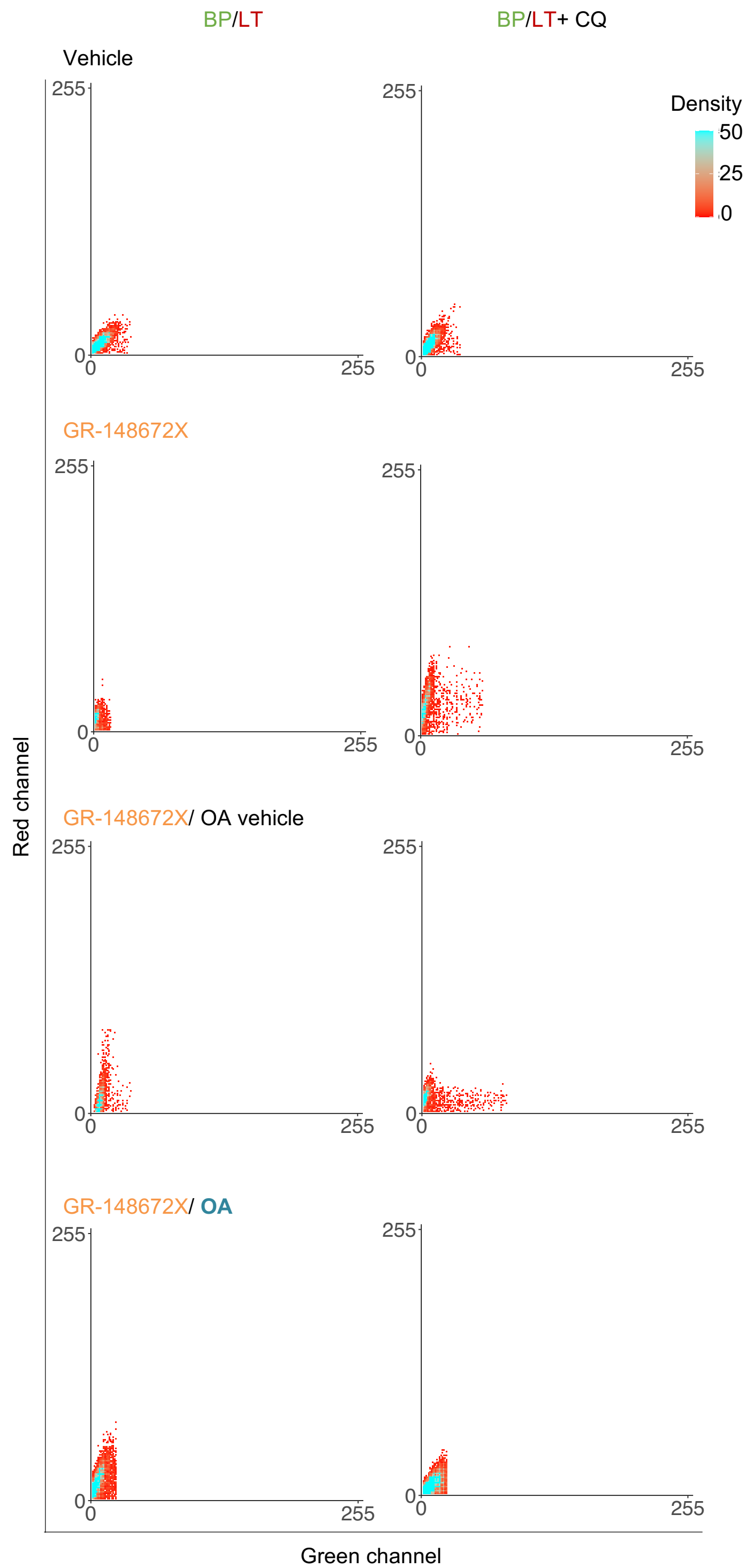

BP/LT
BP/LT+ CQ
Vehicle
GR-148672X
GR-148672X/ OA vehicle
GR-148672X/ OA
Density
50
25
0
255
0
Red channel
Green channel

Figure S4 - Related to Figure 4

A-C] Confocal microscopy individual channel images from Figures 4A-B. **D]** Shown are Coste's masks as a qualitative indication of colocalizing pixels (white) based on calculated thresholds according to Coste's statistical significance algorithm. **E]** 2D correlation histograms (scatter plots) showing the pixel intensity distribution of colocalizing pixels between LDs (green channel, x-axis) and lysosomes (red channel, y-axis). The color scale indicates the pixel density.

SUPPLEMENTARY FIGURE 5

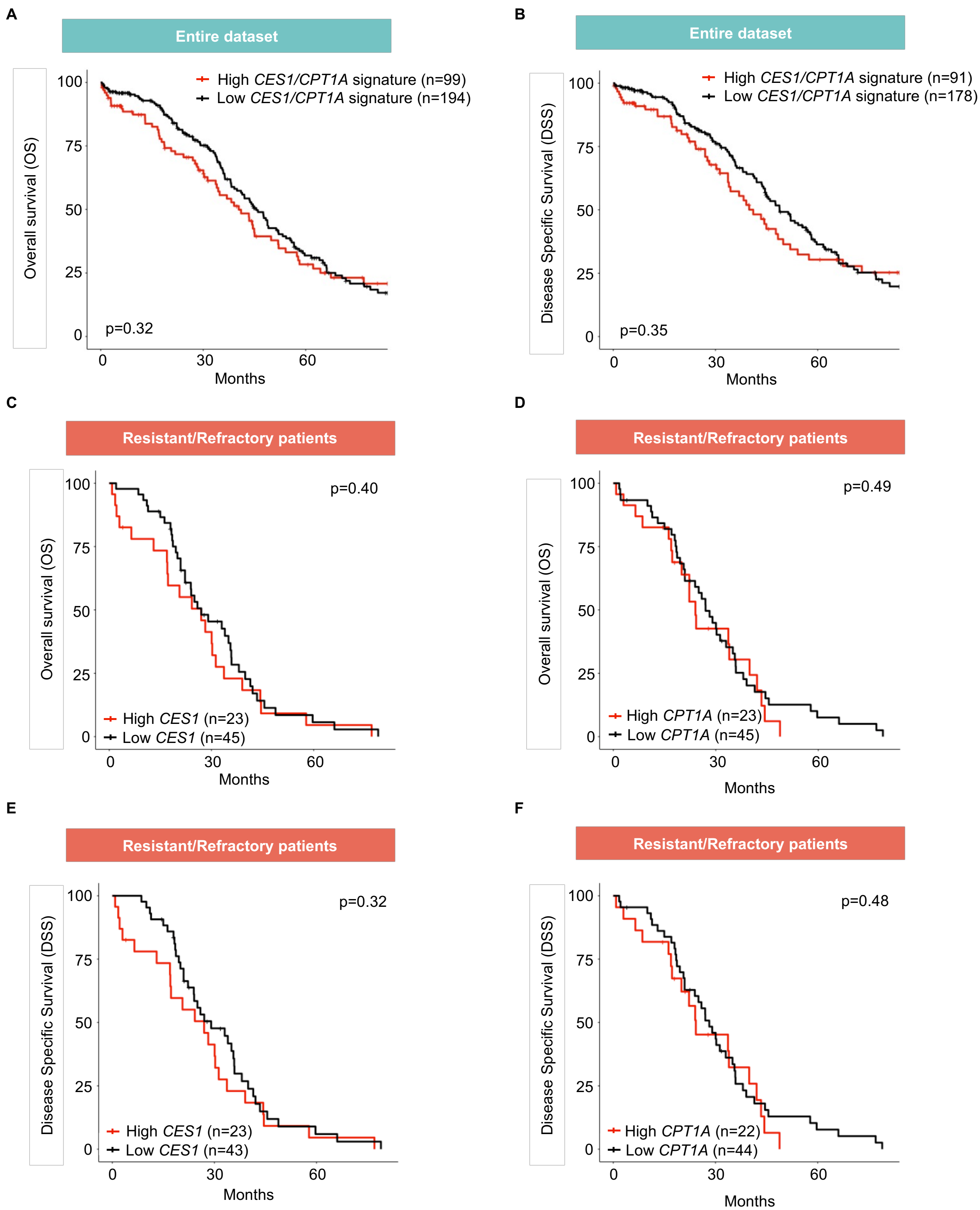

Figure S5 - Related to Figure 6

A-B] Overall Survival (OS) (A, n=293) and Disease-Specific Survival (DSS) (A, n=269) curves for the Entire dataset of patients from the UCSC Toil RNA-Seq recompute dataset from The Cancer Genome Atlas (TCGA) Ovarian (OV) program. Patients were stratified based on the average Z-score of the CES1/CPT1A gene signature, using the first tertile as the threshold. Significance was calculated using the log-rank test. **C-F]** Overall Survival (OS) curves for the chemoresistant/chemorefractory patients, stratified based on CES1 (C, n=68) and CPT1A (D, n=68) expression levels. Disease-Specific Survival (DSS) curves for the chemoresistant/chemorefractory patients, stratified based on CES1 (E, n=66) and CPT1A (F, n=66) expression levels. Patients are from the UCSC Toil RNA-Seq recompute dataset from The Cancer Genome Atlas (TCGA) Ovarian (OV) program and are stratified based on the gene expression levels, using the first tertile as the threshold. Significance was calculated using the log-rank test.
